# NOTCH2 sensitizes the chondrocyte to the inflammatory response of tumor necrosis factor α

**DOI:** 10.1016/j.jbc.2023.105372

**Published:** 2023-10-20

**Authors:** Ernesto Canalis, Jungeun Yu, Vijender Singh, Magda Mocarska, Lauren Schilling

**Affiliations:** 1 Department of Orthopaedic Surgery, UConn Health, Farmington, Connecticut, USA; 2 Department of Medicine, UConn Health, Farmington, Connecticut, USA; 3 UConn Musculoskeletal Institute, UConn Health, Farmington, Connecticut, USA; 4 Computational Biology Core, Institute for System Genomics, UConn, Storrs, Connecticut, USA

**Keywords:** cytokine, Hajdu Cheney syndrome, inflammation, Notch receptor, chondrocyte, tumor necrosis factor α, Interleukin 6

## Abstract

Notch regulates the immune and inflammatory response and has been associated with the pathogenesis of osteoarthritis in humans and preclinical models of the disease. *Notch2*^*tm1.1Ecan*^ mice harbor a NOTCH2 gain-of-function and are sensitized to osteoarthritis, but the mechanisms have not been explored. We examined the effects of tumor necrosis factor α (TNFα) in chondrocytes from *Notch2*^*tm1.1Ecan*^ mice and found that NOTCH2 enhanced the effect of TNFα on *Il6* and *Il1b* expression. Similar results were obtained in cells from a conditional model of NOTCH2 gain-of-function, *Notch2*^*2.1Ecan*^ mice, and following the expression of the NOTCH2 intracellular domain *in vitro*. Recombination signal-binding protein for immunoglobulin Kappa J region partners with the NOTCH2 intracellular domain to activate transcription; in the absence of Notch signaling it inhibits transcription, and *Rbpj* inactivation in chondrocytes resulted in *Il6* induction. Although TNFα induced IL6 to a greater extent in the context of NOTCH2 activation, there was a concomitant inhibition of Notch target genes *Hes1*, *Hey1*, *Hey2*, and *Heyl*. Electrophoretic mobility shift assay demonstrated displacement of recombination signal-binding protein for immunoglobulin Kappa J region from DNA binding sites by TNFα explaining the increased *Il6* expression and the concomitant decrease in Notch target genes. NOTCH2 enhanced the effect of TNFα on NF-κB signaling, and RNA-Seq revealed increased expression of pathways associated with inflammation and the phagosome in NOTCH2 overexpressing cells in the absence and presence of TNFα. Collectively, NOTCH2 has important interactions with TNFα resulting in the enhanced expression of *Il6* and inflammatory pathways in chondrocytes.

Notch receptors (Notch 1–4) are critical determinants of cell differentiation and function in multiple tissues including cartilage (1, 2, 3, 4, 5, 6, 7, 8, 9). Notch receptors are activated following interactions with ligands of the Jagged and Delta-like families. The extracellular domain of Notch is the site of interaction with its ligands, and at the junction of the extracellular and the transmembrane domain rests the negative regulatory region, which is the site of cleavage required for Notch activation (10). Notch ligand interactions lead to the unfolding of the negative regulatory region making it accessible to ADAM metalloproteases and the γ-secretase complex for proteolytic cleavage leading to the release of the Notch intracellular domain (NICD) (11). The NICD translocates to the nucleus where it forms a complex with recombination signal-binding protein for immunoglobulin Kappa J region (RBPJκ) (CSL in human cells) and mastermind-like to induce the transcription of target genes (12, 13, 14, 15). Genes induced by this canonical pathway include members of the Hairy Enhancer of Split (*Hes*) and Hes-related with YRPW motif (*Hey*) families (16, 17, 18). *Notch1*, *2*, *3*, and *4* transcripts are detected in chondrocytes; however, the expression of *Notch2* is significantly greater than that of other Notch receptors in epiphyseal and costal chondrocytes (19)^(E.Canalis, unpublished observations)^. NOTCH2, like all Notch receptors, has its own identity playing a unique function in physiology and disease (8, 20).

Notch has a key regulatory function in the immune and inflammatory response and has been associated with the pathogenesis of osteoarthritis (OA) in humans and preclinical mouse models of the disease (9, 19, 21, 22, 23). Whereas RBPJκ-dependent or canonical Notch signaling is required for cartilage and joint maintenance, sustained supraphysiological activation of Notch is associated with the development of OA and the suppression of chondrogenesis (9, 19, 23, 24, 25). In accordance with these observations, the inactivation of *Rbpj* (encoding RBPJκ) or the Notch target gene *Hes1* prevent the OA that follows the surgical destabilization of the medial meniscus in mice (19, 26).

Our laboratory created and validated a knock-in mouse model harboring a *Notch2*^*6955C>T*^ mutation in exon 34 of *Notch2*, leading to the premature termination of a protein product lacking the PEST domain, which is necessary for the proteasomal degradation of the NOTCH2 NICD; as a consequence the NICD is stable and a gain-of-NOTCH2 function ensues (27). The model, termed *Notch2*^*tm1.1Ecan*^, reproduces many of the functional outcomes of the genetic disorder Hajdu Cheney Syndrome (28, 29, 30, 31, 32). It is of interest that a hallmark of the syndrome is the presence of acroosteolysis associated with inflammation, and *Notch2*^*tm1.1Ecan*^ mice are sensitized to OA and to the osteolytic actions of the inflammatory cytokine tumor necrosis factor α (TNFα) encoded by the *Tnf* gene (28, 33, 34, 35).

NOTCH2 gain-of-function is associated with increased expression of interleukin (IL) 6 in chondrocyte cultures indicating that Notch itself can induce the expression of inflammatory cytokines in cartilage (22, 34, 36). TNFα is a proinflammatory cytokine primarily produced by activated macrophages and known to induce the expression of *Il6* and *Il1b*, but whether TNFα and NOTCH2 interact during the inflammatory response in cartilage tissue is not known (37, 38).

The excessive release of TNFα, IL6, and IL1β during inflammation perturbs joint homeostasis, promotes pathologic bone erosion, and is mechanistically relevant to the development of OA, and interactions of these cytokines with Notch signaling could play a key role in the inflammatory response in cartilage tissue. Consequently, we asked the question as to whether a NOTCH2 gain-of-function not only sensitizes mice to OA but also to the inflammatory response to TNFα in chondrocytes. To this end, we examined the effects of TNFα in chondrocytes from *Notch2*^*tm1.1Ecan*^ mutant mice and additional models of NOTCH2 gain-of-function and explored mechanisms responsible for an enhanced inflammatory response to TNFα in the context of NOTCH2 activation.

## Results

### Enhanced Notch2 signaling inhibits chondrogenesis

**Figure 1:**
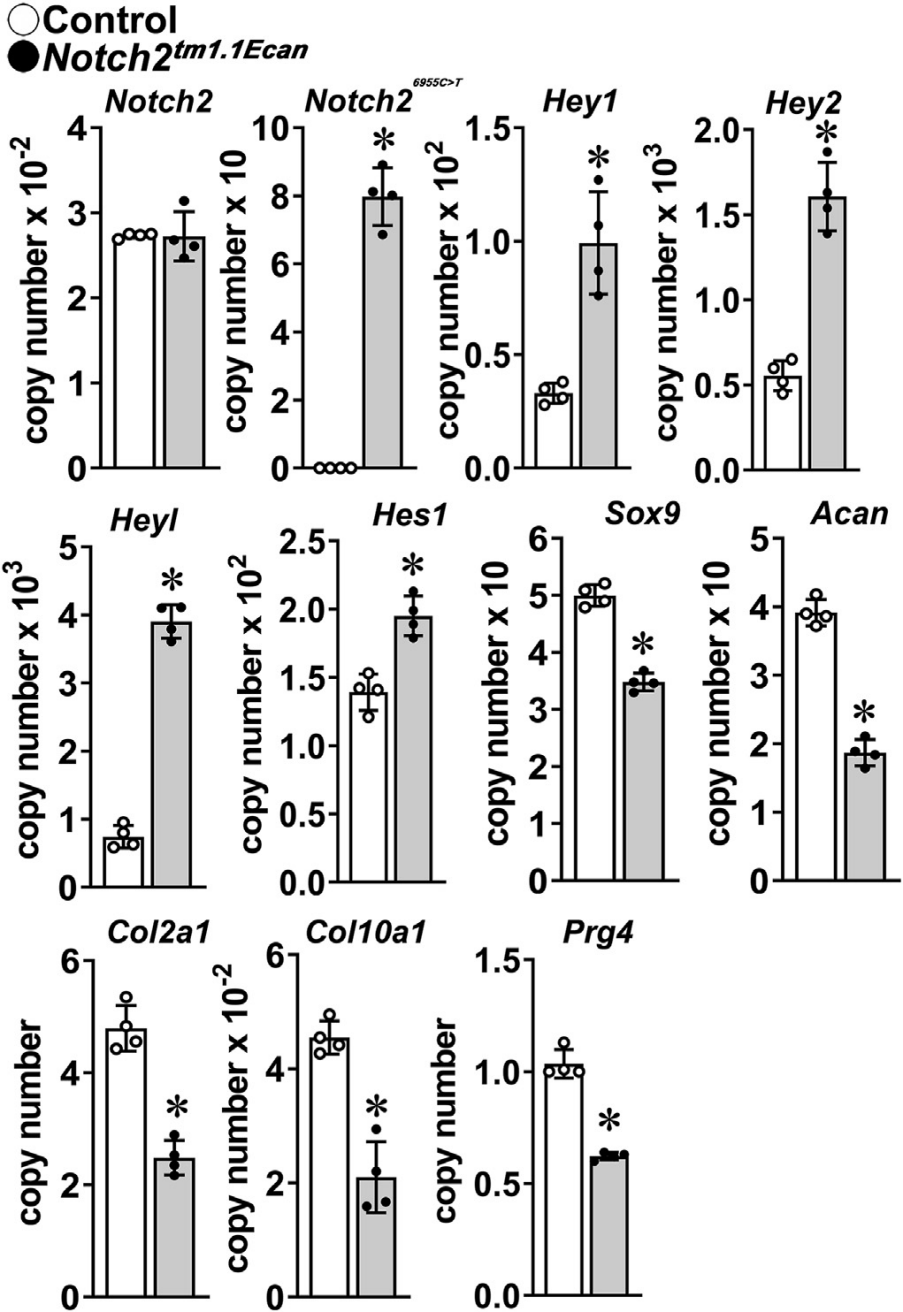
**Enhanced NOTCH2 signaling inhibits chondrogenesis.** Chondrocyte-enriched cells were isolated from newborn heterozygous *Notch2*^*tm1.1Ecan*^ mice (*closed circles*, *gray bars*) and wildtype (*open circles*, *white bars*) littermate controls and cultured for 3 days and gene expression measured by qRT-PCR. Data are expressed as *Notch2*, *Notch2*^*6955C>T*^, *Hey1*, *Hey2*, *Heyl*, *Hes1*, *Sox9*, *Acan*, *Col2a1*, *Col10a1*, and *Prg4* copy number corrected for *Rpl38*. Values are means (bars) ± SD and individual determinations (dots); n = 4. ∗Significantly different between control and *Notch2*^*tm1.1Ecan*^, *p* < 0.05 by unpaired *t* test. qRT-PCR, quantitative reverse transcription-polymerase chain reaction.

In an initial experiment, chondrocytes from the epiphysis of 3- to 4-day-old heterozygous *Notch2*^*tm1.1Ecan*^ mice and littermate controls were isolated and cultured in monolayer without further expansion. Heterozygous *Notch2*^*tm1.1Ecan*^ mice were used because in previous work, we found that the homozygous mutation is lethal during development or immediately after birth (27). *Notch2*^*6955C>T*^ transcripts were expressed exclusively in *Notch2*^*tm1.1Ecan*^ cells, where there was a concomitant induction of the canonical Notch target genes *Hey1*, *2*, *l*, and *Hes1* documenting enhanced Notch signal inactivation (Fig. 1). The transcript expression of the chondrogenic markers *Sox9*, *Acan* (encoding aggrecan), *Col2a1*, and *Col10a1*, as well as the expression of *Prg4* (encoding lubricin) was decreased in *Notch2*^*tm1.1Ecan*^ cells indicating that the *Notch2*^*tm1.1Ecan*^ mutation suppressed chondrogenesis, confirming that Notch activation inhibits chondrocyte differentiation (24, 25).

### Notch2^tm1.1Ecan^ chondrocytes are sensitized to the actions of TNFα and IL1β on the inflammatory response

**Figure 2:**
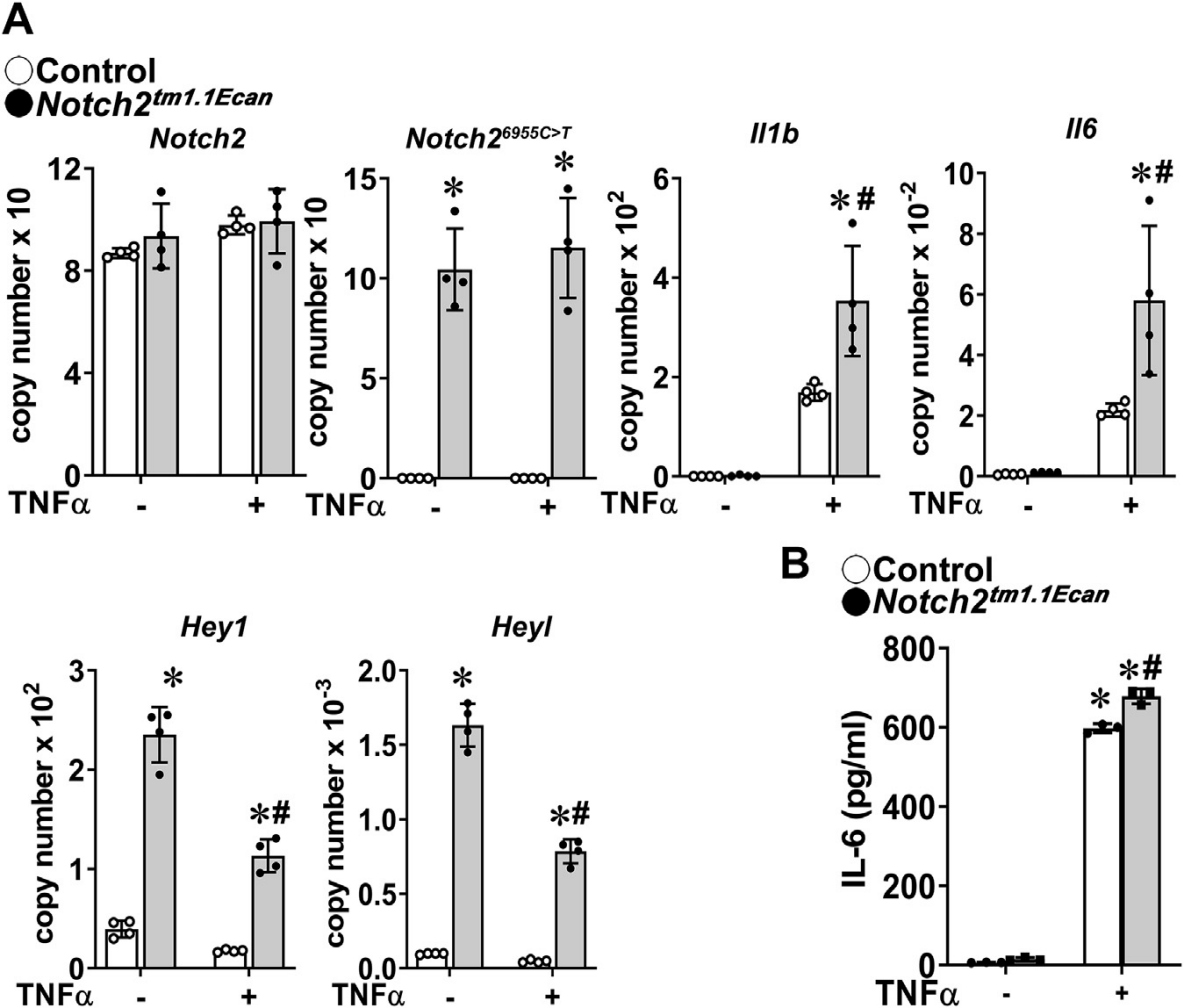
***Notch2***^***tm1.1Ecan***^**(*Notch2***^***6955C>T***^**) mutant chondrocytes are sensitized to the action of TNFα on the inflammatory response.***A* and *B*, chondrocyte-enriched cells from newborn heterozygous *Notch2*^*tm1.1Ecan*^ mice (*closed circles*, *gray bars*) and control littermates (*open circles*, *white bars*) were cultured to confluence, transferred, and in *Panel A* exposed to TNFα at 50 ng/ml or vehicle for 6 h in the absence of serum and mRNA expression determined by qRT-PCR or in *Panel B* exposed to TNFα for 24 h for IL6 determinations by ELISA. Data for mRNA are expressed as *Notch2*, *Notch2*^*6955C>T*^, *Il1b*, *Il6*, *Hey1*, and *Heyl* copy number corrected for *Rpl38*. IL6 concentrations are expressed in pg/ml. Values are means (bars) ± SD and individual determinations (dots); n = 4 for all data sets. Significantly different between: ∗control and *Notch2*^*tm1.1Ecan*^; ^#^TNFα and vehicle, both *p* < 0.05 by two-way ANOVA with post-hoc analysis by Tukey. qRT-PCR, quantitative reverse transcription-polymerase chain reaction; TNFα, tumor necrosis factor α.

**Figure 3:**
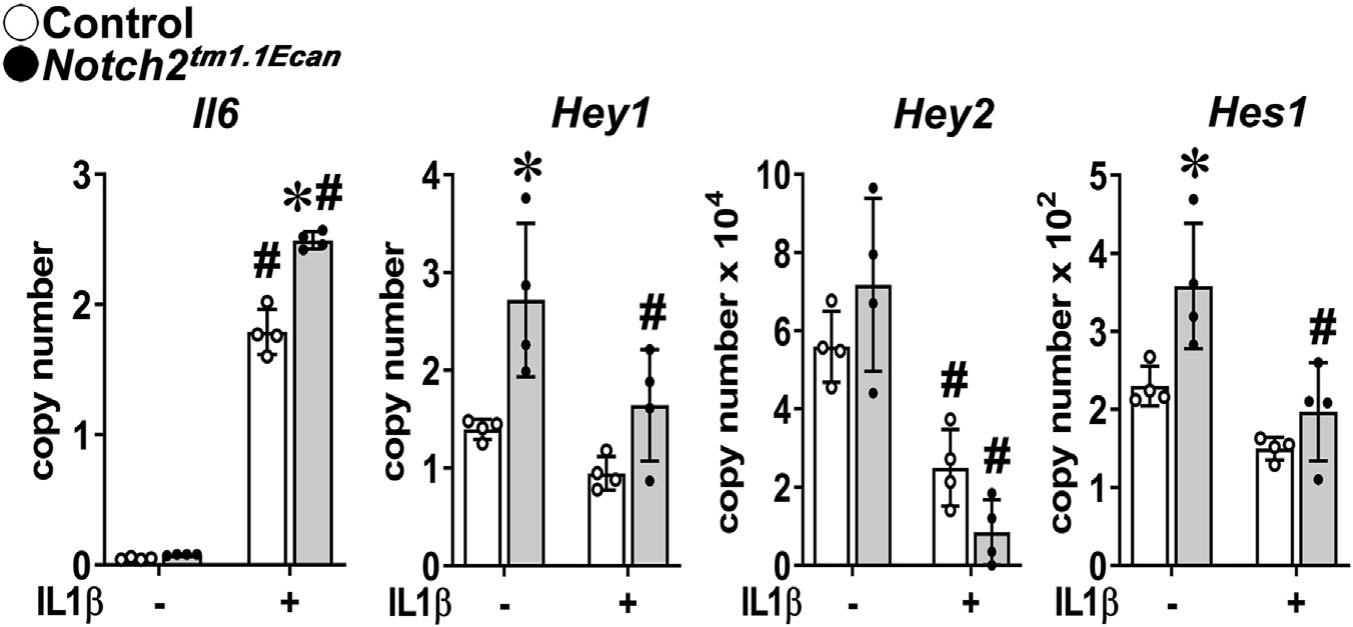
***Notch2***^***tm1.1Ecan***^**(*Notch2***^***6955C>T***^**) mutant chondrocytes are sensitized to the action of IL1β on the inflammatory response.** Chondrocyte-enriched cells from newborn *Notch2*^*tm1.1Ecan*^ mice (*closed circles*, *gray bars*) and control littermates (*open circles*, *white bars*) were cultured to confluence, transferred, and exposed to Il1β at 10 ng/ml or vehicle for 6 h in the absence of serum and mRNA expression determined by qRT-PCR. Data are expressed as *Il6*, *Hey1*, *Hey2* and *Hes1* copy number corrected for *Rpl38*. Values are means (bars) ± SD and individual determinations (dots); n = 4. Significantly different between: ∗*Notch2*^*tm1.1Ecan*^ and control, ^#^IL1β and vehicle, both *p* < 0.05 by two-way ANOVA with post-hoc analysis by Tukey. qRT-PCR, quantitative reverse transcription-polymerase chain reaction.

To examine whether the *Notch2*^*tm1.1Ecan*^ mutation sensitizes chondrocytes to the actions of TNFα, chondrocytes from heterozygous *Notch2* mutant mice and control littermates were treated with TNFα or vehicle for 6 h. TNFα induced the expression of *Il6* and *Il1b*, and the effect was amplified significantly in *Notch2*^*tm1.1Ecan*^ mutant cells (Fig. 2). Whereas canonical Notch target genes were induced in *Notch2*^*tm1.1Ecan*^ mutant cells, TNFα decreased the expression of *Hey1* and *Heyl* suggesting that the enhanced expression of *Il6* and *Il1b* by the *Notch2* mutation was not directly related to an amplification of Notch canonical signaling. TNFα had no effect on the expression of *Notch2*^*6991C>T*^ and *Notch2*. TNFα increased immunoreactive IL6 levels in chondrocytes, and the effect was amplified in *Notch2*^*tm1.1Ecan*^ mutant cells (Fig. 2*B*). These findings were substantiated by testing the effects of IL1β. Confirming the observations with TNFα, IL1β induced *Il6* to a greater extent in *Notch2*^*tm1.1Ecan*^ chondrocytes than in control cells, while it suppressed the expression of the Notch canonical targets *Hes1*, *Hey1*, and *Hey2* (Fig. 3).

### The inflammatory response to TNFα is enhanced in chondrocytes from Notch2^tm2.1Ecan^ conditional mice

**Figure 4:**
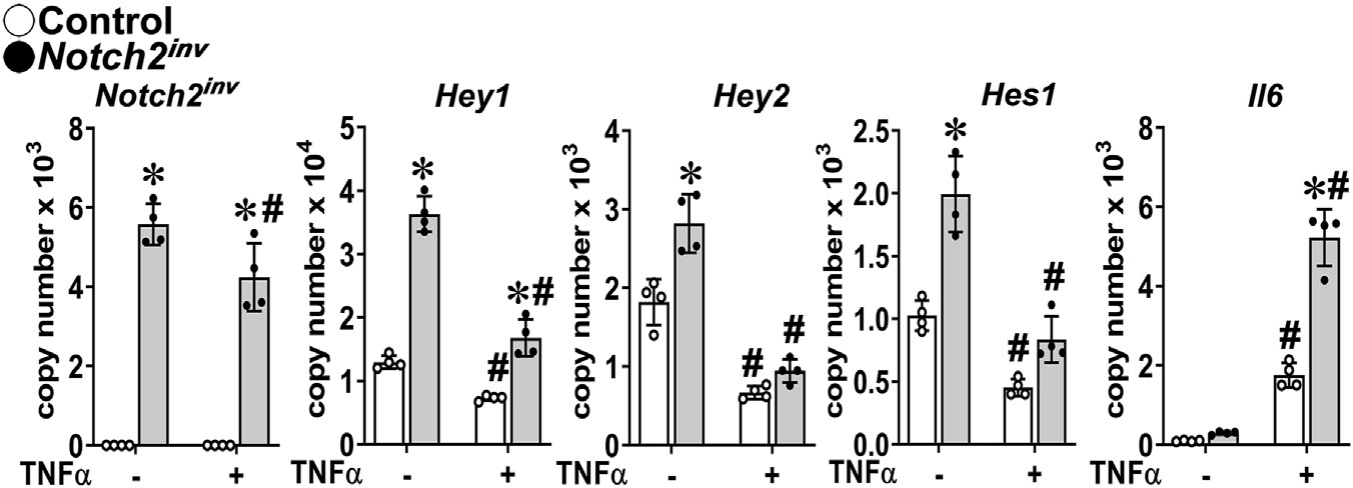
**NOTCH2 overexpression sensitizes chondrocytes to the action of TNFα on the inflammatory response.** Chondrocyte-enriched cells from newborn homozygous *Notch2*^*tm2.1Ecan*^ mice were cultured to ∼70% confluence and transduced with Ad-CMV-Cre (*closed circles*; *gray bars*) to invert the COIN module or Ad-CMV-GFP (*open circles*, *white bars*) as a control and cultured for 48 h and exposed to TNFα at 50 ng/ml for 6 h or vehicle in the absence of serum and mRNA expression determined by qRT-PCR. Data are expressed as *Notch2*^*ΔPEST*^ or *Notch2*^*INV*^, *Hey1*, *Hey2*, *Hes1*, and *Il6* copy number corrected for *Rpl38*. Values are means (*bars*) ± SD and individual determinations (*circles*); n = 4. Significantly different between ∗*Notch2*^*INV*^ and control; ^#^TNFα and control, *p* < 0.05 by two-way ANOVA with post-hoc analysis by Tukey. CMV, cytomegalovirus; COIN, conditional by inversion; GFP, green fluorescent protein; qRT-PCR, quantitative reverse transcription-polymerase chain reaction; TNFα, tumor necrosis factor α.

To validate the observations in *Notch2*^*tm1.1Ecan*^ mice, chondrocytes from the *Notch2*^*tm2.1Ecan*^ (*Notch2*^*COIN*^) conditional mouse model of Hajdu Cheney Syndrome were obtained (39). In this model, Cre-mediated recombination results in the introduction of a STOP codon upstream of sequences coding for the PEST domain and in the translation of a truncated and stable NOTCH2 protein. As a consequence, a NOTCH2 gain-of-function analogous to the one observed in *Notch2*^*tm1.1Ecan*^ global mutant mice ensues. Cultures from homozygous *Notch2*^*tm2.1Ecan*^ mice were infected with an adenoviral vector expressing Cre recombinase under the control of the cytomegalovirus (CMV) promoter (Ad-CMV-Cre), and parallel cultures infected with an adenoviral vector where the CMV promoter governs green fluorescent protein (GFP) expression (Ad-CMV-GFP) served as controls. Ad-CMV-Cre, but not Ad-CMV-GFP, infection led to the inversion of the conditional by inversion (*COIN*) module and expression of the *Notch2*^*ΔPEST*^ or *Notch2*^*ΔINV*^ mRNA with the consequent induction of *Hes1*, *Hey1*, and *Hey2* demonstrating activation of Notch signaling (Fig. 4). In accordance with the results observed in *Notch2*^*tm1.1Ecan*^ mutant mice, TNFα induced *Il6* transcripts to a greater extent in chondrocytes from *Notch2*^*tm2.1Ecan*^ conditional mice following inversion of the COIN module than in control cells. In addition and in agreement with results in *Notch2*^*tm1.1Ecan*^ mice, TNFα suppressed the induced *Hes1*, *Hey1*, and *Hey2* in *Notch2*^*tm2.1Ecan*^ cells following the inversion of the COIN module and introduction of the STOP codon; a modest decrease in *Notch2*^*INV*^ transcripts was observed.

### The NOTCH2 NICD is responsible for the enhanced inflammatory response to TNFα

**Figure 5:**
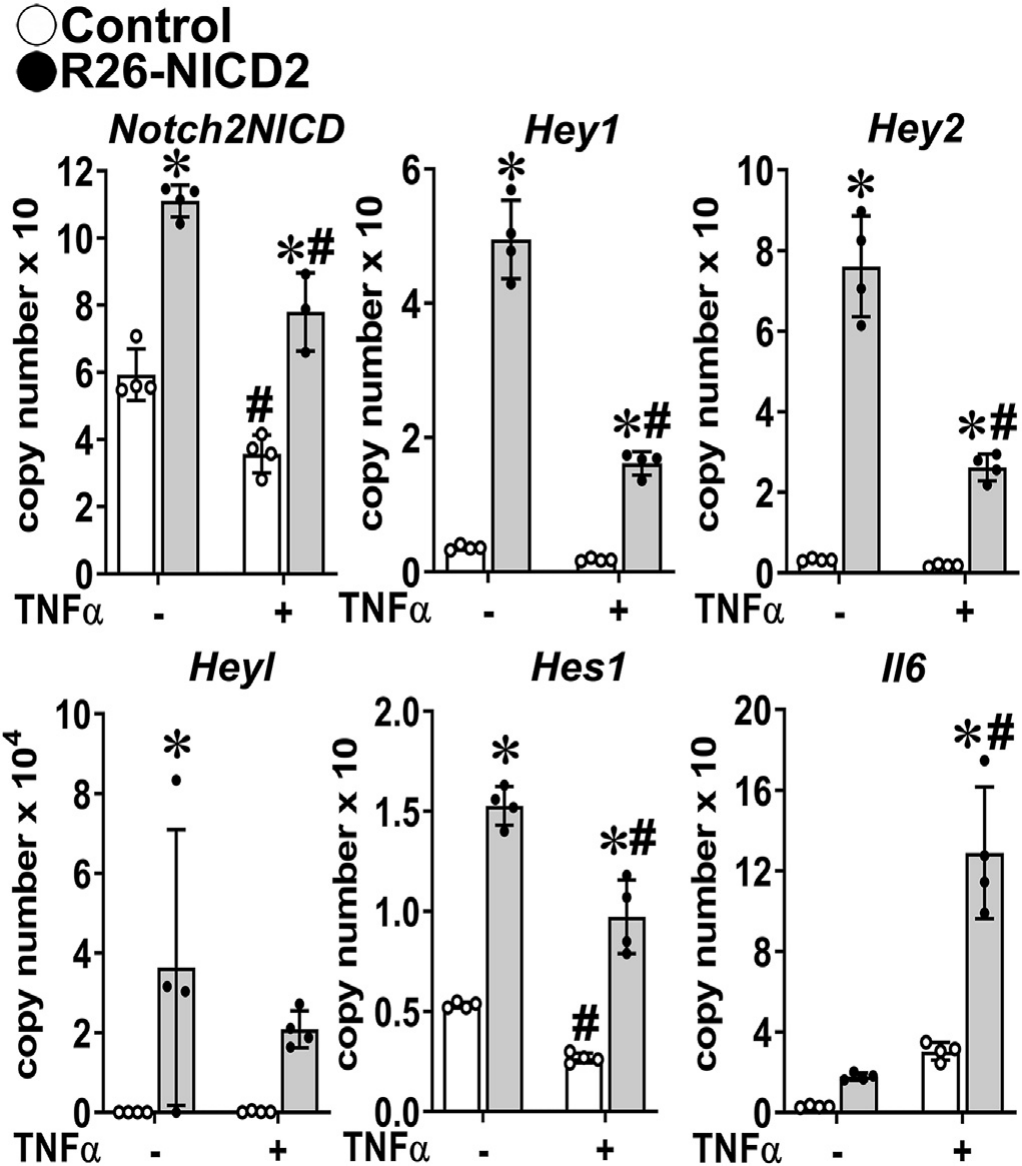
**NOTCH2 NICD overexpression sensitizes chondrocytes to the action of TNFα on the inflammatory response.** Chondrocyte-enriched cells from newborn *R26-NICD2* mice were cultured to ∼70% confluence and transduced with Ad-CMV-Cre (*black dots*, *gray bars*) to induce NOTCH2 NICD or Ad-CMV-GFP (*open circles*, *white bars*) as a control and cultured for 48 h and exposed to TNFα at 50 ng/ml for 6 h or vehicle in the absence of serum and mRNA expression determined by qRT-PCR. Data are expressed as *Notch2*^*ΔPEST*^, *Hey1*, *Hey2*, *Heyl Hes1* and *Il6* copy number corrected for *Rpl38*. Values are means (*bars*) ± SD and individual determinations (*circles*); n = 4. Significantly different between: ∗R26-NICD2 and control; ^#^TNFα and control, *p* < 0.05 by two-way ANOVA with post-hoc analysis by Tukey. CMV, cytomegalovirus; GFP, green fluorescent protein; NICD, Notch intracellular domain; qRT-PCR, quantitative reverse transcription-polymerase chain reaction; TNFα, tumor necrosis factor α.

To test whether the NOTCH2 NICD was directly responsible for the NOTCH2–TNFα interactions and augmentation of the IL6 response to TNFα, chondrocytes were obtained from homozygous and heterozygous R26-NICD2 mice. In this model, sequences coding for the NOTCH2 NICD are cloned into the *Rosa26* locus downstream of a neo-STOP cassette flanked by *loxP* sequences. Upon Cre recombination, the STOP cassette is excised, and the NOTCH2 NICD expressed under the control of *Rosa26*. Chondrocyte-enriched cells from heterozygous (shown) and homozygous (not shown) R26-NICD2 mice were infected with Ad-CMV-Cre to induce the NOTCH2 NICD or with Ad-CMV-GFP to serve as controls. Analysis of mRNA levels by quantitative reverse transcription-polymerase chain reaction (qRT-PCR) revealed that activation of Notch signaling induced a significant increase in *Hes1*, *Hey1*, *Hey2*, and *Heyl* mRNA levels, and the induction was tempered in TNFα-treated cells (Fig. 5). TNFα also caused a decrease in *Notch2NICD* expression. Confirming results obtained from *Notch2*^*tm1.1Ecan*^ and *Notch2*^*tm2.1Ecan*^ inverted chondrocytes, TNFα induced *Il6* to a greater extent in NOTCH2 NICD expressing than in control cells demonstrating that direct activity of the NICD was responsible for the *Il6* amplification of the TNFα effect in chondrocytes. The response to TNFα was similar to cells from heterozygous and homozygous R26-NICD2 mice transduced with Ad-CMV-Cre.

### RBPJκ is a suppressor of Il6 expression

**Figure 6:**
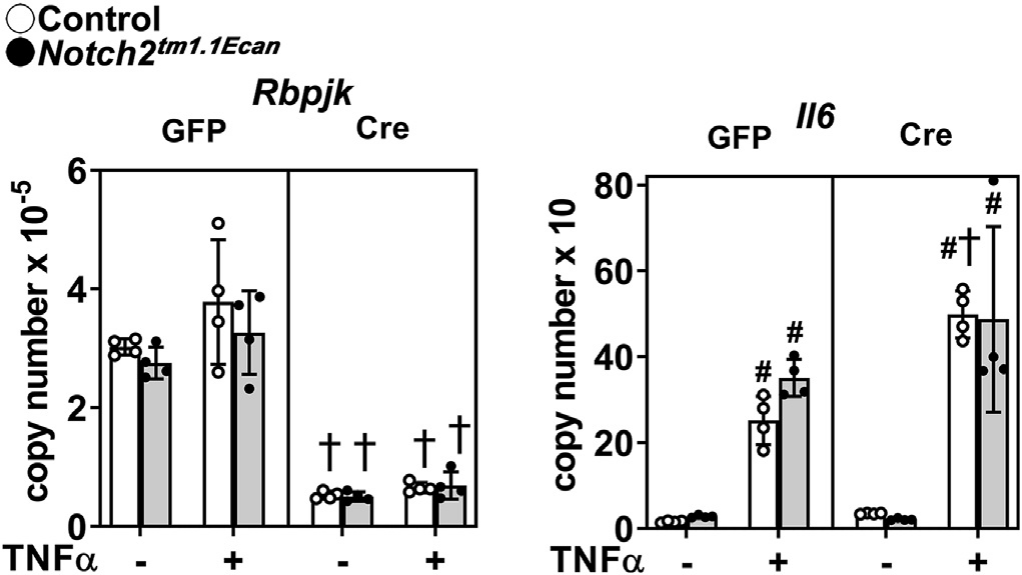
**RBPJκ is an inhibitor of *Il6* expression.** Chondrocyte-enriched cells from newborn *Notch2*^*tm1.1Ecan*^*;Rbpj*^*loxP/loxP*^ mice were cultured to ∼70% confluence and transduced with Ad-CMV-Cre (*closed circles*, *gray bars*) to recombine *loxP* flanked sequences or Ad-CMV-GFP (*open circles*, *white bars*) as a control and then cultured for 48 additional hours and exposed to TNFα at 50 ng/ml for 6 h or vehicle in the absence of serum and mRNA expression determined by qRT-PCR. Data are expressed as *Rbpj* and *Il6* copy number corrected for *Rpl38*. Values are means (*bars*) ± SD and individual determinations (*dots*); n = 4. Significantly different between: ^#^TNFα and control; ^+^Ad-Cre and Ad-GFP, all *p* < 0.05 by three-way ANOVA with post-hoc analysis by Tukey. CMV, cytomegalovirus; GFP, green fluorescent protein; qRT-PCR, quantitative reverse transcription-polymerase chain reaction; RBPJκ, recombination signal-binding protein for immunoglobulin Kappa J region; TNFα, tumor necrosis factor α.

To determine the contributions of Notch canonical signaling to the interactions between NOTCH2 and TNFα, *Notch2*^*tm1.1Ecan*^ mice were backcrossed into a homozygous *Rbpj*^*loxP/loxP*^ background. Chondrocytes from *Notch2*^*tm1.1Ecan*^;*Rbpj*^*loxP/loxP*^ mice were infected with Ad-CMV-Cre viral particles to delete *loxP* flanked sequences and inactivate *Rbpj*^*loxP/loxP*^;Ad-CMV-GFP–infected cells served as a control. Infection with Ad-CMV-Cre resulted in the deletion of *Rbpj* and the loss of the stimulatory effect of the NOTCH2 gain-of-function on the Notch target genes *Hes1*, *Hey1*, and *Hey2* (not shown) since RBPJκ is required for their induction by Notch. The deletion of *Rbpj* resulted in an increase in the expression of *Il6* in both vehicle (*p* > 0.05) and TNFα-treated cultures by ∼2-fold demonstrating that RBPJκ is an inhibitor of *Il6* expression (Fig. 6). No amplification was observed in *Notch2*^*tm1.1Ecan*^ chondrocytes in the context of the *Rbpj* deletion. This suggests that the NOTCH2 gain-of-function acts by converting RBPJκ from an inhibitor to a stimulator of transcription, and no further stimulation can be achieved by NOTCH2 in the absence of RBPJκ.

To explore further the mechanisms responsible for the enhancement of the TNFα effect on *Il6* expression and possible interactions between RBPJκ and *Il6* transcription and concomitant inhibition of Notch signaling, electrophoretic mobility shift assay (EMSA) was carried out in chondrocyte-enriched cells harvested from *Notch2*^*tm1.1Ecan*^ and control wildtype littermates. A biotinylated oligonucleotide containing the consensus sequence for *Rbpj* (*CSL*) was bound by nuclear protein extracts from control and *Notch2*^*tm1.1Ecan*^ cells, and an excess of unlabeled *Rbpj* oligonucleotides prevented this interaction whereas an excess mutant *Rbpj* unlabeled oligonucleotide did not, demonstrating the specificity of the protein-DNA interaction (Fig. S1). There was increased binding of nuclear extracts from *Notch2*^*tm1.1Ecan*^ to biotinylated *Rbpj* confirming the formation of a larger or more stable RBPJκ complex in the context of enhanced Notch canonical signaling by the NOTCH2 gain-of-function. TNFα suppressed the formation of nuclear protein complexes with the biotinylated *Rbpj* consensus oligonucleotide in *Notch2*^*tm1.1Ecan*^ and control cells supporting the notion that TNFα prevents the interaction of RBPJκ with DNA. Since the *Rpbj* inactivation experiments indicate that RBPJκ is an inhibitor of *Il6* transcription, its displacement by TNFα from DNA binding sites would contribute to the enhanced expression of *Il6* by TNFα.

### TNFα induces NF-κB signal activation to a greater extent in Notch2^tm1.1Ecan^ chondrocytes

**Figure 7:**
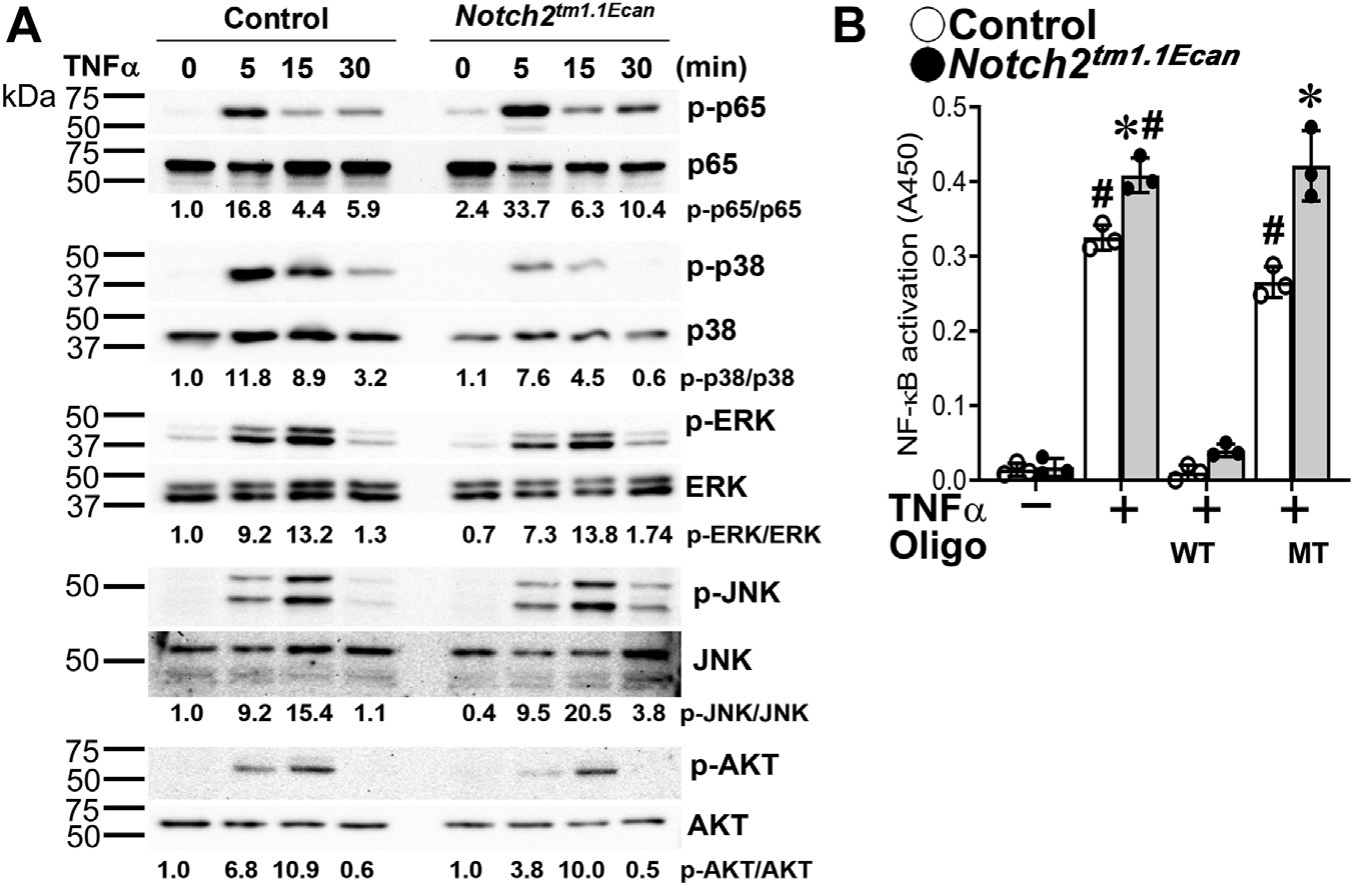
**TNFα-induced NF-κB signal activation is enhanced modestly in *Notch2***^***tm1.1Ecan***^**mutant cells.***In Panel A*, chondrocyte-enriched cells from newborn *Notch2*^*tm1.1Ecan*^ mice and control littermates were cultured to confluence, transferred, and exposed to TNFα at 200 ng/ml or vehicle in the absence of serum for the indicated periods of time. Whole cell lysates (35 μg of total protein) were examined by immunoblotting using anti-p-p65, p-p38, p-ERK, p-JNK, and p-AKT antibodies, stripped, and reprobed with anti-p65, p38, ERK, JNK, and AKT antibodies. The band intensity was quantified by ImageLab software (version 5.2.1), and the numerical ratios of phosphorylated/unphosphorylated signal determined and shown under each blot. Control values for phosphorylated and unphosphorylated protein ratios at time 0 are both normalized to 1. *In Panel B*, chondrocytes from *Notch2*^*tm1.1Ecan*^ mice (*gray bars*, *black dots*) and control littermates (*white bars*, *open circles*) were exposed to TNFα at 200 ng/ml in the absence of serum for 1 h, and 20 μg of nuclear extracts from each sample were examined by TransAM Flexi NF-κB p65 activation assay kit in the presence and absence of a wildtype (WT) or mutant (MT) competitor in 10x-fold excess and colorimetric changes were measured at 450 nm. Values are means (*bars*) ± SD and individual determinations (*dots*); n = 3, technical replicates. Significantly different between: ^∗^control and TNFα; ^#^control and *Notch2*^*tm1.1Ecan*^, all *p* < 0.05 by two-way ANOVA with post-hoc analysis by Tukey.

To explore further signaling pathways responsible for the enhanced inflammatory response in *Notch2*^*tm1.1Ecan*^ chondrocytes, cells from mutant and control littermates were treated with TNFα 200 ng/ml for up to 30 min and cell extracts analyzed by Western blot. TNFα induced the phosphorylation of mitogen-activated protein kinases, ERK and JNK to a similar extent in control and mutant cells and p38 phosphorylation was diminished in *Notch2*^*tm1.1Ecan*^ cells. In contrast, p65 phosphorylation was enhanced in *Notch2*^*tm1.1Ecan*^ cells (Fig. 7*A*). In addition, TNFα induced NF-κB transactivation in chondrocytes from both genotypes and the effect was amplified in cells from *Notch2*^*tm1.1Ecan*^ mice indicating enhanced NF-κB activation by the NOTCH2 gain-of-function (Fig. 7*B*).

### Mechanisms responsible for the NOTCH2–TNFα interactions

**Figure 8:**
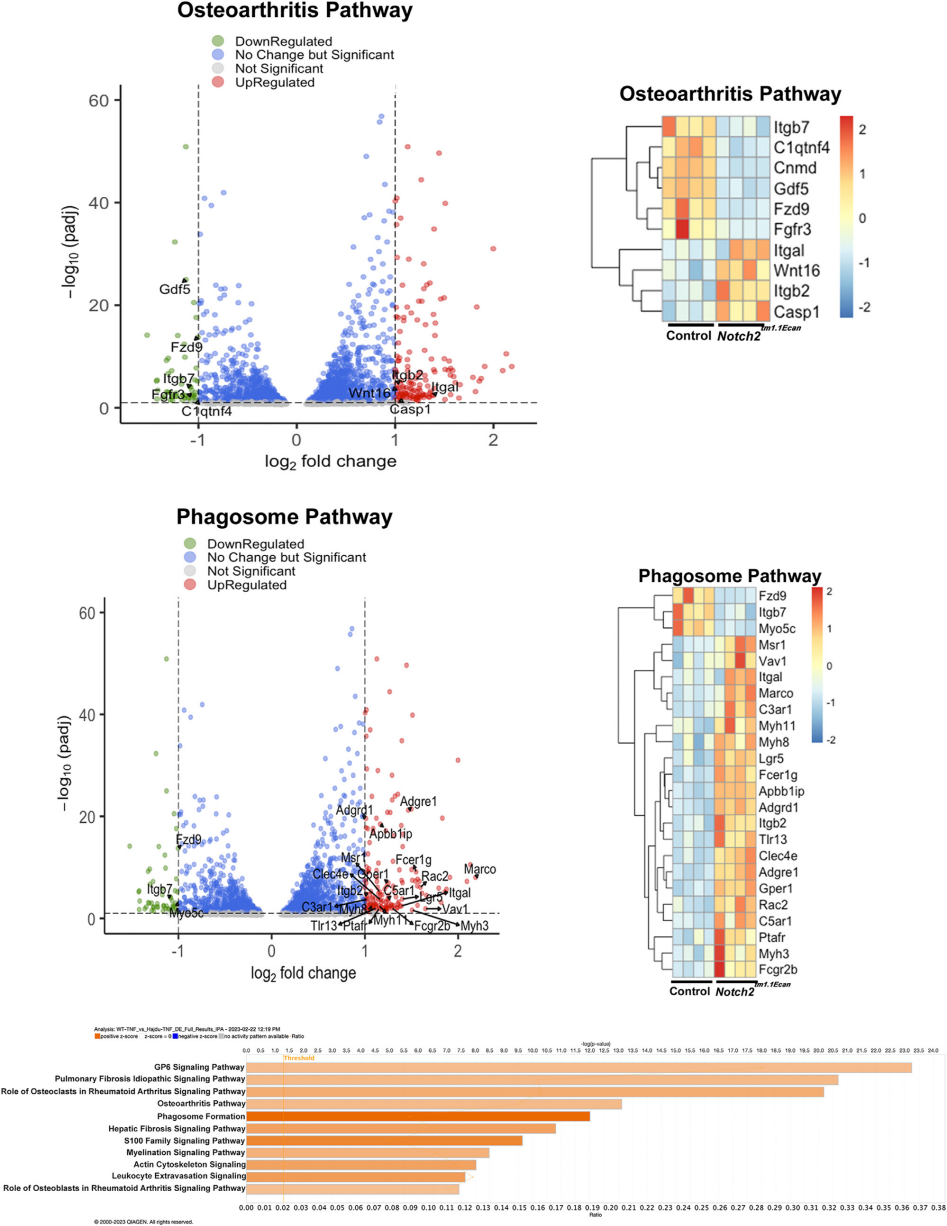
**The phagosome and inflammatory response are enhanced in *Notch2***^***tm1.1Ecan***^**chondrocytes.** Chondrocyte-enriched cells from newborn *Notch2*^*tm1.1Ecan*^ mice and control littermates were cultured to confluence and treated with vehicle or TNFα at 50 ng/ml for 6 h in the absence of serum. Cells were collected for total RNA extraction and analyzed by RNA-Seq. The volcano plots reveal differentially regulated genes at log 2fc1 p adjusted value of 0.1 highlighting genes regulated in osteoarthritis and phagosome formation following ingenuity pathway analysis (IPA). *Bar graph* indicates select signaling pathways positively affected by *Notch2*^*tm1.1Ecan*^*versus* control chondrocytes, both treated with TNFα; n = 4, analyzed by IPA. Heat map of differentially expressed genes between *Notch2*^*tm1.1Ecan*^ and control chondrocytes, both treated with TNFα, log 2fc1 p adjusted value 0.1 affected by osteoarthritis pathway and phagosome formation as determined by IPA.

To understand the molecular mechanisms associated with the effect of NOTCH2 on the amplification of the response to TNFα, RNA from *Notch2*^*tm1.1Ecan*^ and control chondrocytes was examined by RNA-Seq analysis. There were 208 differentially regulated genes between *Notch2*^*tm1.1Ecan*^ and control chondrocytes treated with TNFα (Fig. 8; and Table S1). Ingenuity Pathway Analysis revealed that genes associated with the inflammatory response, including pathogen-induced cytokine storm signaling, OA pathway and role of osteoblasts and osteoclasts in rheumatoid arthritis signaling, as well as genes associated with the phagosome formation pathway, were enhanced in *Notch2*^*tm1.1Ecan*^ chondrocytes compared to control, both treated with TNFα (Fig. 8). A similar pattern of signal activation was observed in the absence of TNFα treatment (not shown). Venn diagrams revealed that of the 208 differentially expressed genes between *Notch2*^*tm1.1Ecan*^ and control chondrocytes, 10 genes were associated with the OA pathway and 25 with the phagosome formation pathway (Fig. S2). Further analysis of differentially regulated genes by qRT-PCR demonstrated downregulation of *Gdf5* and *Fgfr3*, genes associated with articular cartilage development and joint integrity, by the *Notch2*^*tm1.1Ecan*^ mutation in chondrocytes (Fig. S3) (40, 41, 42). *Casp1*, a gene associated with OA and inflammation, and *Marco* (Fig. S3), *Vav1*, *Fcerlg* and *Adgre1* (not shown), genes associated with the phagosome pathway were induced by TNFα, but no further induction was observed in *Notch2*^*tm1.1Ecan*^ mutant chondrocytes (43, 44, 45, 46). *Rac2* a gene associated with the phagosome pathway, was induced by TNFα to a greater extent in *Notch2*^*tm1.1Ecan*^ chondrocytes than in control cells (Fig. S3) (47).

## Discussion

Previous work demonstrated that a NOTCH2 gain-of-function sensitizes mice to the development of arthritis following destabilization of the medial meniscus surgeries (34). The present work extends those observations and explores possible mechanisms responsible for the enhanced inflammatory response in the context of the NOTCH2 gain-of-function. We demonstrated that NOTCH2 has a pronounced inhibitory effect on chondrocyte differentiation confirming previous observations demonstrating an inhibitory role of Notch signaling in chondrogenesis (24).

We found that NOTCH2 interacts with TNFα and IL1β enhancing their effect on the induction of *Il6* transcripts. The amplification of the TNFα effect was observed in cells from two models of NOTCH2 gain-of-function, namely a global *Notch2*^*tm1.1Ecan*^ and a conditional (COIN) *Notch2*^*tm2.1Ecan*^ mutant mouse model harboring mutations that result in the expression of a stable NOTCH2 NICD devoid of the PEST domain, and a gain-of-Notch function. The amplification of the TNFα response was secondary to direct effects of the NOTCH2 NICD since it was also observed in cells from R26-NICD2 mice overexpressing the NOTCH2 NICD under the control of the *Rosa26* locus. The induction of *Il6* by NOTCH2 is in agreement with the stimulatory effects of NOTCH1 on *Il6* expression in chondrocytes and supports the notion that activation of Notch signaling in cartilage contributes to the inflammatory response and possibly to OA progression (9, 36).

Recently, we reported that serum levels of TNFα are not different between *Notch2*^*tm1.1Ecan*^ and control mice (33). In addition, when the serum from *Notch2*^*tm1.1Ecan*^ mice was examined by Proteome Profiler Mouse Cytokine Array it revealed no difference in the levels of proinflammatory cytokines in the systemic circulation of *Notch2*^*tm1.1Ecan*^ mice when compared to control serum. However, levels of inflammatory cytokines increase during aging and in the context of NOTCH2 one would expect an amplified response to these cytokines and this may play an important role in the inflammatory response in cartilage tissue during aging (48, 49, 50, 51).

The inactivation of *Rbpj* enhanced the expression of *Il6* to levels similar to those observed with the NOTCH2 gain-of-function in *Notch2*^*tm1.1Ecan*^ cells. The enhanced expression of *Il6* in *Rbpj* deleted cells would indicate that RBPJκ is an inhibitor of *Il6* transcription. The results are also consistent with the notion that when the NOTCH2 NICD forms a complex with RBPJκ it displaces co-repressors so that RPBJκ is no longer an inhibitor of *Il6* transcription and *Il6* is induced. This is in accordance with the fact that RBPJκ is a transcriptional repressor that induces gene expression only following interactions with the NICD, so that its downregulation could result in the induction of *Il6* and lead to cellular changes analogous to those associated with the activation of Notch signaling (7, 12). Indeed, the induction of *Il6* by TNFα was of a similar magnitude in *Rbpj* deleted cells from control and *Notch2*^*tm1.1Ecan*^ mice. It is possible that the levels of *Il6* mRNA were maximal and no further induction could be achieved in the presence of NOTCH2. Because the *Rbpj* deletion enhanced the expression of *Il6*, it is not possible to conclude from the experiments that the effect observed with the NOTCH2 gain-of-function was dependent, or not, on Notch canonical signaling. It is of interest that the deletion of *Rbpj* in the limb bud during development or in articular chondrocytes in postnatal life causes severe OA, and this could possibly be related to enhanced expression of *Il6*, as shown in the present work (23, 52). Indeed, IL6 plays a fundamental role in the OA that develops following the destabilization of medial meniscus surgeries (53).

NOTCH2 overexpression caused induction of Notch target genes of the *Hes* and *Hey* families. Concomitant to the TNFα dependent induction of *Il6*, there was a decrease in the expression of *Hes1* and *Hey* genes in control and NOTCH2 overexpressing chondrocytes. *Heyl* expression in chondrocytes is low and at times undetectable. EMSA revealed a decrease in the RBPJκ–nuclear protein complex by TNFα possibly contributing to the decreased expression of Notch canonical target genes. This displacement of RBPJκ from DNA binding sites may mimic the consequences of the *Rbpj* inactivation and explain the induction of *Il6*. An additional mechanism that may operate in the downregulation of Notch target genes by IL6 is a decrease in the expression of *Notch2* observed in 2 out of 3 experiments. Previous work revealed a critical function of HES1 in OA progression, and HES1 can induce *Il6* expression in chondrocytes (26). But it is not likely that HES1 is responsible for the actions of NOTCH2 since *Hes1* transcripts were decreased by TNFα at a time that *Il6* was induced, and the effect amplified by NOTCH2.

TNFα induced NF-κB activation, and this effect was enhanced by NOTCH2, possibly contributing to the induction of *Il6* in the context of the NOTCH2 gain-of-function. Similar interactions have been reported between NOTCH2 and NF-κB signaling in cells of the myeloid/osteoclast lineage (54). Following phosphorylation, p65 is degraded in the nucleus by ubiquitination, and we find that unphosphorylated p65 levels were decreased by the NOTCH2 gain-of-function (55). This is possibly because of an increase in NF-κB activation by NOTCH2 or enhanced ubiquitination by Notch signaling as it has been reported for other nuclear proteins (56, 57). The decrease in p65 may also be related to an upregulation of the phagosome pathway by NOTCH2, similar to the one reported for chaperone-mediated autophagy (58).

RNA-Seq analysis revealed that NOTCH2 induces pathways associated with OA and the inflammatory response and upregulates the phagosome pathway. It is possible that NOTCH2–TNFα interactions influence a subpopulation of CD163-expressing phagocytic chondrocytes or direct the differentiation of articular chondrocytes toward the phagocytic pathway (59, 60). Activation of these pathways may explain the enhanced inflammatory response induced by NOTCH2 as well as the propensity to OA in mouse models of NOTCH2 gain-of-function. Indeed, further analysis by qRT-PCR revealed downregulation of *Gdf5* and *Fgfr3* in *Notch2*^*tm1.1Ecan*^ chondrocytes, and these genes play an important function in cartilage development and structure and are associated with OA (40, 41, 42). qT-PCR also demonstrated amplification of the *Rac2* response to TNFα by the NOTCH2 gain-of-function and *Rac2* is associated with the phagosome pathway (47).

In conclusion, NOTCH2 amplifies the inflammatory response to TNFα in chondrocytes, and TNFα modulates interactions of RBPJκ with gene regulatory sequences.

## Experimental procedures

### Genetically modified mice

*Notch*^*tm1.1Ecan*^ mice harboring a 6955C > T substitution in the *Notch2* locus have been characterized in previous studies and were backcrossed into a C57BL/6 background for ≥8 generations (27). *Notch2*^*tm2.1Ecan*^ or *Notch2*^*COIN*^ mice, backcrossed into a C57BL/6 background, were previously characterized and were designed to introduce a STOP codon in exon 34 of *Notch2* upstream of sequences coding for the PEST domain, following Cre-mediated recombination of a COIN module (39). R26-NICD2 mice were created by Ryuichi Nishinakamura (Kumamoto University) and kindly provided by Fanxin Long in a C57BL/6 background (61, 62). In R26-NICD2 mice, sequences coding for the NOTCH2 NICD are cloned into the *Rosa26* locus downstream a neo-STOP cassette flanked by *loxP* sequences so that the NOTCH2 NICD is expressed under the control of *Rosa26* following the excision of the cassette by Cre recombination. *Rbpj*^*tm1Hon*^ or *Rbpj*^*loxP/loxP*^ mice were obtained from Riken (RBRC1071) and backcrossed into a C57BL/6 background (63). In these mice, *loxP* sites are inserted upstream of exon 6 and downstream of exon 7 of *Rbpj*. To determine whether Notch canonical signaling is responsible for the effects of NOTCH2, *Notch2*^*tm1.1Ecan*^ mutant mice were backcrossed into a homozygous *Rbpj*^*loxP/loxP*^ conditional background (*Notch2*^*tm1.1Ecan*^*;Rbpj*^*loxP/loxP*^).

Genotyping was conducted in tail DNA extracts by PCR using specific primers from Integrated DNA technologies (IDT) (Table 1). All animal experiments were approved by the Institutional Animal Care and Use Committee of UConn Health.

**Table 1:** Primers used for genotyping

Allele	Strand	Sequence 5′-3′	Amplicon size (bp)
*Notch2*^*COIN*^	Forward Reverse	5′-CCGGGCCGCGACTGAAACCCTAG-3′ 5′-CCACCACCTCCAGGAGTTGGGC-3′	330
*Notch2*^*tm1.1Ecan*^	Forward Reverse	5′-CCCTTCTCTCTGTGCGGTAG-3′ 5′-CTCAGAGCCAAAGCCTCACTG-3′	WT = 308 *Notch2*^*tm1.1Ecan*^ = 403
*Notch2*^*WT*^	Forward Reverse	5′-GCTCAGACCATTGTGCCAACCTAT-3′ 5′-CAGCAGCATTTGAGGAGGCGTAA-3′	100
*Rpbj*^*loxP*^	Forward Reverse WT Reverse	5′-GTTCTTAACCTGTTGGTCGGAACC-3′ 5′-GCAATCCATCTTGTTCAATGGCC-3′ 5′-GCTTGAGGCTTGATGTTCTGTATTGC-3′	WT = 500 Flox = 610
*R26-NICD2*	Forward Reverse WT Forward WT Reverse	5′-AAGGGACTGGCTGCTATTGG-3′ 5′-ATATCACGGGTAGCCAACGC-3′ 5′-CTCTCCCAAAGTCGCTCTG-3′ 5′-TACTCCGAGGCGGATCACAAGC-3′	WT = 224 Rosa = 420

### Chondrocyte cultures

Chondrocyte-enriched cells were isolated from the epiphyses of long bones of the hind and fore limbs from 3-to 4-day-old *Notch2*^*tm1.1Ecan*^ mice and control littermates or from *Notch2*^*tm2.1Ecan*^ (*Notch2*^*COIN*^), R26-NICD2 or *Notch2*^*tm1.1Ecan*^;*Rbpj*^*loxP/loxP*^ mice. Surrounding tissues were dissected under a Unitron Z850 stereo microscope, and epiphyseal cartilage collected in high glucose Dulbecco’s modified Eagle’s medium (DMEM, Life Technologies), as described (64). The tissue was exposed to 0.25% trypsin, 0.9 mM EDTA (Life Technologies) and subsequently to 200 U/ml of collagenase type II (Worthington Biochemical Corporation) at 37 °C. Digested cartilage was strained through a 70 μm membrane, and cells are collected by centrifugation and cultured in DMEM supplemented with 10% heat inactivated fetal bovine serum (Atlanta Biologicals) at 37 °C in a humidified 5% CO_2_ incubator (25, 34).

To invert the COIN module and introduce a STOP codon into exon 34 upstream of the PEST domain of *Notch2* in *Notch2*^*tm2.1Ecan*^ cells to induce NOTCH2 NICD in R26-NICD2 cells or to delete *Rbpj* sequences in cells from *Rbpj*^*loxP/loxP*^ mice, chondrocytes were transferred to DMEM in the absence of serum for 1 h and exposed overnight to 300 to 600 multiplicity of infection of replication-defective recombinant adenoviruses. An adenoviral vector expressing Cre recombinase under the control of the CMV promoter (Ad-CMV-Cre, Vector Biolabs) was used to invert the COIN module in *Notch2*^*tm2.1Ecan*^ cells to excise the STOP cassette in R26-NICD2 cells or to excise *Rbpj* sequences in *Rbpj*^*loxP/loxP*^ cells. An adenoviral vector where the CMV promoter directs expression of GFP (Ad-CMV-GFP, Vector Biolabs) was used as control. Following infection, chondrocyte-enriched cells were allowed to recover for 24 to 48 h and cultured in the presence of DMEM containing 10% fetal bovine serum and exposed to test agents as indicated in text and legends (36).

### Quantitative reverse transcription-PCR

Total RNA was extracted from chondrocytes with the RNeasy Mini kit (Qiagen), in accordance with manufacturer’s instructions. The integrity of the RNA was assessed in random samples by microfluidic electrophoresis on an Experion system (Bio-Rad), and RNA with a quality indicator number equal to or higher than 7.0 was used for subsequent analysis. Equal amounts of RNA were reverse-transcribed using the iScript RT-PCR kit (Bio-Rad) and amplified in the presence of specific primers (Table 2) (all from IDT) with the SsoAdvanced Universal SYBR Green Supermix (Bio-Rad) at 60 °C for 40 cycles. Transcript copy number was estimated by comparison with a serial dilution of cDNA for *Acan*, *Il6*, *Illb*, *Notch2*, *Col10a1*, *Sox9*, and *Rbpj* (from Thermo Fisher Scientific), *Hes1*, *Col2a1*, and *Rpl38* (American Type Culture Collection), *Prg4* (Bioscience), *Hey1* and *Hey2* (T. Iso, Gunma University) and *Heyl* (D. Srivastava, Gladstone Institute of Cardiovascular Disease or Dharmacon, Horizon Discovery) (65, 66).

**Table 2:** Primers used for qRT-PCR determinations

Gene	Strand	Sequence	GenBank accession number
*Acan*	Fwd	5′-ATGGTCCTTCTATGACATACACTCCCCG-3′	NM_007242
	Rev	5′-TTGTTACAGCGCCACCAAGG-3′	
*Casp1*	Fwd	5′-ATGAATACAACCACTCGTA-3′	NM_009807.2
	Rev	5′-TTCTCTGAGGTCAACTTG-3′	
*Col2a1*	Fwd	5′-GACCCAAACACTTTCCAACCGCAGT-3′	NM_031163 NM_003396
	Rev	5′-TCATCAGGTCAGGTCAGCCATT-3′	
*Col10a1*	Fwd	5′-CAGGCTTTCTGGGATGCCGCTTGT-3′	NM_009925
	Rev	5′-GGGCACCTACTGCTGGGTAA-3′	
*Fgfr3*	Fwd	5′-GAAGAATGGCAAAGAATT-3′	NM_001163215.2
	Rev	5′-TCAACTACACAGGTATAG-3′	
*Gdf5*	Fwd	5′-GTCAGGAAGCAGAGGTA-3′	NM_008109.4
	Rev	5′-CGTAAGATCCGCAGTTC-3′	
*Hes1*	Fwd	5′-ACCAAAGACGGCCTCTGAGCACAGAAAGT-3′	NM_008235
	Rev	5′-ATTCTTGCCCTTCGCCTCTT-3′	
*Hey1*	Fwd	5′-ATCTCAACAACTACGCATCCCAGC-3′	NM_010423
	Rev	5′-GTGTGGGTGATGTCCGAAGG-3′	
*Hey2*	Fwd	5′-AGCGAGAACAATTACCCTGGGCAC-3′	NM_013904
	Rev	5′-GGTAGTTGTCGGTGAATTGGACCT-3′	
*Heyl*	Fwd	5′-CAGTAGCCTTTCTGAATTGCGAC-3′	NM_013905
	Rev	5′-AGCTTGGAGGAGCCCTGTTTC-3′	
*Illb*	Fwd	5′-GGACAGAATATCAACCAACAAGTG-3′	NM_008361
	Rev	5′-TCGTTGCTTGGTTCTCCTT-3′	
*Il6*	Fwd	5′-CGGCCTTCCCTACTTCACAAGTCCG-3′	NM_00314054; NM_031168
	Rev	5′-CAGGTCTGTTGGGAGTGGTATCC-3′	
*Marco*	Fwd	5′-CCGTCAGCAGTTCAACAACCT-3′	NM_010766.3
	Rev	5′-TGGAGAGCCTCGTTCACCTT-3′	
*Notch2*	Fwd	5′-TGACGTTGATGAGTGTATCTCCAAGCC-3′	NM_010928
	Rev	5′-GTAGCTGCCCTGAGTGTTGTGG-3′	
*Notch2*^*ΔPEST*^	Fwd	5′-GGCTTTCCCACCTACCAT-3′	No Applicable
	Rev	5′-TAGTCGGGCACGTCGTAG'	
*Prg4*	Fwd	5′-CGCCTTTTCCAAAGATCAATACTA-3′	NM_021400.3 NM_001110146
	Rev	5′-GTGGTAATTGCTCTTGCTGTT-3′	
*Rac2*	Fwd	5′-GGCGGATGTTCATAG-3′	NM_009008.3
	Rev	5′-TCTGTATGAGGATGGAT-3′	
*Rbpj*	Fwd	5′-GAACTTGGAAGGGAAGAACTACTG-3′	NM_001080927.2
	Rev	5′-GTCATCGCTGTTGCCATAGAA-3′	
*Rpl38*	Fwd	5′-AGAACAAGGATAATGTGAAGTTCAAGGTTC-3′	NM_001048057; NM_001048058; NM_023372
	Rev	5′-CTGCTTCAGCTTCTCTGCCTTT-3′	
*Sox9*	Fwd	5′-CCTACTACAGTCACGCAGCCG-3′	NM_011448
	Rev	5′-GGGTTCATGTAAGTGAAGGTGGA-3′	

GenBank accession numbers identify transcript recognized by primer pairs.

The level of *Notch2*^*6955C>T*^ mutant transcript was measured as described previously (27). Total RNA was reverse transcribed with Moloney murine leukemia virus reverse transcriptase in the presence of reverse primers for *Notch2* (5′-GGATCTGGTACATAGAG-3′) and *Rpl38* (Table 2). *Notch2* cDNA was amplified by PCR in the presence of TaqMan gene expression assay mix, including specific primers (5′-CATCGTGACTTTCCA-3′ and 5′-GGATCTGGTACATAGAG-3′) and a 6-carboxyfluorexcein-labeled DNA probe of sequence 5′-CATTGCCTAGGCAGC-3′ covalently attached to a 3′-minor groove binder quencher (Thermo Fisher Scientific), and SsoAdvanced Universal Probes Supermix (Bio-Rad) at 60 °C for 45 cycles (67). *Notch2*^*6955C>T*^ transcript copy number was estimated by comparison to a serial dilution of a synthetic DNA fragment (IDT) containing ∼200 bp surrounding the 6955C > T mutation in the *Notch2* locus, and cloned into pcDNA3.1 (Thermo Fisher Scientific) by isothermal single reaction assembly using commercially available reagents (New England Biolabs) (68).

The primers used to detect *Notch2* allow for the detection of *Notch2* and *Notch2*^*COIN*^ but not *Notch2*^*ΔPEST*^ or *Notch2*^*INV*^ transcripts (39). To monitor for the efficiency of the COIN inversion, *Notch2*^*ΔPEST*^ or *Notch2*^*INV*^ transcripts were detected with primers that generate an amplicon straddling the artificial splice junction created within exon 34 of the targeted *Notch2* locus upon inversion of the COIN module. These primers do not recognize wildtype *Notch2* or *Notch2*^*COIN*^ mRNA prior to *COIN* inversion. *Notch2*^*ΔPEST*^ copy number was estimated by comparison with a serial dilution of an ∼200 bp synthetic DNA template (IDT) cloned into pcDNA3.1 (Thermo Fisher Scientific) by isothermal single reaction assembly using commercially available reagents (New England Biolabs).

Amplification reactions were conducted in CFX96 qRT-PCR detection systems (Bio-Rad), and fluorescence was monitored at the end of the elongation step during every PCR cycle. Data are expressed as copy number corrected for *Rpl38* expression estimated by comparison with a serial dilution of cDNA for *Rpl38* (69). Data for *Gdf5*, *Fgfr3*, *Casp1*, *Rac2*, and *Marco* are expressed as relative values corrected for *Rpl38* expression.

### Enzyme-linked immunosorbent assay

To determine whether the *Notch2*^*tm1.1Ecan*^ mutation induces a change in Il6 protein levels, confluent *Notch2*^*tm1.1Ecan*^ chondrocyte-enriched cells were cultured for 3 days before exposure for 24 h to DMEM in the absence or presence of TNFα. Il6 concentrations in the medium were measured with a mouse Il6 ELISA kit, in accordance with manufacturer's instructions (BD Biosciences).

### Illumina transcriptome library preparation and RNA sequencing

Total RNA was quantified, and purity ratios determined using a NanoDrop 2000 spectrophotometer (Thermo Fisher Scientific), and RNA quality was assessed on an Agilent TapeStation 4200 (Agilent Technologies) with the RNA High Sensitivity assay. Only samples with ribosomal integrity numbers values above 9.0 were used for library preparation. Total RNA was processed for mRNA-sequencing using the Illumina TruSeq Stranded mRNA Sample Preparation kit following the manufacturer’s protocol (Illumina). Libraries were validated for length and adapter dimer removal using the Agilent TapeStation 4200 D1000 High Sensitivity assay (Agilent Technologies), and then they were quantified and normalized using the dsDNA High Sensitivity Assay for Qubit 3.0 (Thermo Fisher Scientific). Libraries were prepared for Illumina sequencing by denaturing and diluting the libraries per manufacturer’s protocol (Illumina). All samples were multiplexed pooled into one sequencing pool, equally normalized, and run as one sample pool across the Illumina NextSeq 500, version 2.5 kit. Target read depth was achieved for each sample with paired end 75 bp reads. Raw reads were trimmed with Trimmomatic (Version 0.39), with a quality threshold of 30 and length threshold of 45 and mapped to Mus Musculus genome (GRCm39 ensembl release 105) with HISAT2 (version 2.2.1) (70). The resulting SAM files were converted into BAM format using samtools (version 1.9). The counts were generated against the features with htseq-count (version 0.11.0) (71). The differential expression of genes between conditions was evaluated using DESeq2 (72). Covariates were introduced in the DESeq2 analysis to increase the accuracy of results, and genes showing less than 10 counts across the compared samples were excluded from analysis. Genes with a false discovery rate <0.05 or <0.1 were considered significant and used in the downstream analysis. The processed RNA-seq results were further analyzed using Ingenuity Pathway Analysis.

### Electrophoretic mobility shift assay

Nuclear extracts were obtained from chondrocytes of *Notch2*^*tm1.1Ecan*^ and littermate controls treated with vehicle or TNFα. A double-stranded DNA oligonucleotide containing the *CSL* (*Rbpj*) consensus sequence found in the Epstein-Barr virus nuclear antigen 2 promoter (forward strand sequence: 5′-GGAAACACGCCGTGGGAAAAAATTTGGG-3′) biotinylated on both 5′-termini was synthesized commercially (IDT) (73). Binding reactions of nuclear extracts with biotinylated DNA at a concentration of 1 μM were carried out with the LightShift Chemiluminescent EMSA Kit, as recommended by the manufacturer (Thermo Fisher Scientific) (74). To determine the specificity of the interactions between the nuclear extracts and the biotinylated oligonucleotides, unlabeled homologous or mutant DNA was added in 200-fold excess. Nucleic acid–protein complexes were resolved on nondenaturing, nonreducing 4% polyacrylamide gels for 45 min and subsequently transferred to a nylon membrane with a 0.45 μm pore size (MP Biomedicals) for 30 min at 4 °C, and crosslinking of the transferred complexes at 120 mJ/cm^2^ for 1 min using a CL-1000 UV-light crosslinking instrument (UVP). The biotinylated DNA was detected with a streptavidin-horseradish peroxidase (HRP) conjugate following manufacturer’s instructions for the LightShift Chemiluminescent Kit detection module, and images of chemiluminescence reactions were acquired with a Chemidoc XSR molecular imager (Bio-Rad).

### Immunoblotting

Cells from control and experimental mice were extracted in buffer containing 25 mM Tris-HCl (pH 7.5), 150 mM NaCl, 5% glycerol, 1 mM EDTA, 0.5% Triton X-100, 1 mM sodium orthovanadate, 10 mM NaF, 1 mM phenyl methyl sulfonyl fluoride, and a protease inhibitor cocktail (all from Sigma Aldrich). Total cell lysates (35 μg of total protein) were separated by sodium dodecyl sulfate–polyacrylamide gel electrophoresis in 8 or 10% polyacrylamide gels and transferred to Immobilon-P membranes (Millipore). The blots were probed with anti-p-p38 (9211), p38 (9212), p-ERK (9101), ERK (9102), p-JNK (4668), JNK (9252), p-AKT (9271), AKT (9272), p-p65 (3033), and p65 (8242) antibodies (all from Cell Signaling Technology). The blots were exposed to anti-rabbit IgG conjugated to HRP (Sigma-Aldrich) and incubated with a chemiluminescence detection reagent (Bio-Rad). Chemiluminescence was detected by ChemiDoc XSR+ molecular imager (Bio-Rad) with Image Lab software (version 5.2.1), and the amount of protein in individual bands was quantified (33, 75).

### NF-κB activation assay

TNFα-treated chondrocytes from control or experimental mice were lysed prior to nuclear extraction using the Nuclear Extract Kit (Active Motif, Inc). To detect and quantify NF-κB activation, 20 μg of nuclear extract samples were examined using a commercial enzyme-linked immunosorbent assay-based kit (TransAM Flexi NF-κB p65, Active Motif, Inc) in accordance with manufacturer’s instructions (76). Briefly, nuclear extracts were incubated with a biotinylated consensus NF-κB binding sequence (5′-GGGACTTTCC-3′) (1 pmol/well) and the reaction mixture transferred into assay wells. Samples were incubated with anti- NF-κB p65 antibody and anti-rabbit IgG conjugated to HRP and colorimetric changes measured in an iMark Microplate Absorbance Reader (Bio-Rad) at 450 nm with a reference wavelength of 655 nm. To assess the specificity of NF-κB binding to the biotinylated probe, unlabeled wildtype, or mutated consensus NF-κB binding oligonucleotides were added in excess (10 pmol/well) to the reaction mixture.

### Statistics

Data are expressed as means ± SD. Statistical differences were determined by Student’s *t* test, two-way, or three-way ANOVA analysis of variance with Tukey test for multiple comparisons.

## Data availability

RNA Seq data have been uploaded and can be viewed in GEO (https://www.ncbi.nlm.nih.gov/geo/query/acc.cgi?acc=GSE224255).

## Supporting information

This article contains supporting information.

## Conflict of interest

The authors declare no conflicts of interest with the contents of this article.

## References

[1] Bai S., Kopan R., Zou W., Hilton M.J., Ong C.T., Long F. (2008). NOTCH1 regulates osteoclastogenesis directly in osteoclast precursors and indirectly via osteoblast lineage cells. J. Biol. Chem..

[2] Engin F., Yao Z., Yang T., Zhou G., Bertin T., Jiang M.M. (2008). Dimorphic effects of Notch signaling in bone homeostasis. Nat. Med..

[3] Hilton M.J., Tu X., Wu X., Bai S., Zhao H., Kobayashi T. (2008). Notch signaling maintains bone marrow mesenchymal progenitors by suppressing osteoblast differentiation. Nat. Med..

[4] Zanotti S., Smerdel-Ramoya A., Stadmeyer L., Durant D., Radtke F., Canalis E. (2008). Notch inhibits osteoblast differentiation and causes osteopenia. Endocrinology.

[5] Yu J., Canalis E. (2020). Notch and the regulation of osteoclast differentiation and function. Bone.

[6] Canalis E., Parker K., Feng J.Q., Zanotti S. (2013). Osteoblast lineage-specific effects of notch activation in the skeleton. Endocrinology.

[7] Siebel C., Lendahl U. (2017). Notch signaling in development, tissue homeostasis, and disease. Physiol. Rev..

[8] Canalis E. (2018). Notch in skeletal physiology and disease. Osteoporos. Int..

[9] Liu Z., Chen J., Mirando A.J., Wang C., Zuscik M.J., O'Keefe R.J. (2015). A dual role for NOTCH signaling in joint cartilage maintenance and osteoarthritis. Sci. Signal..

[10] Sanchez-Irizarry C., Carpenter A.C., Weng A.P., Pear W.S., Aster J.C., Blacklow S.C. (2004). Notch subunit heterodimerization and prevention of ligand-independent proteolytic activation depend, respectively, on a novel domain and the LNR repeats. Mol. Cell. Biol..

[11] Gordon W.R., Zimmerman B., He L., Miles L.J., Huang J., Tiyanont K. (2015). Mechanical allostery: evidence for a force requirement in the proteolytic activation of notch. Dev. Cell.

[12] Kovall R.A. (2008). More complicated than it looks: assembly of Notch pathway transcription complexes. Oncogene.

[13] Nam Y., Sliz P., Song L., Aster J.C., Blacklow S.C. (2006). Structural basis for cooperativity in recruitment of MAML coactivators to Notch transcription complexes. Cell.

[14] Schroeter E.H., Kisslinger J.A., Kopan R. (1998). Notch-1 signalling requires ligand-induced proteolytic release of intracellular domain. Nature.

[15] Wilson J.J., Kovall R.A. (2006). Crystal structure of the CSL-Notch-Mastermind ternary complex bound to DNA. Cell.

[16] Iso T., Kedes L., Hamamori Y. (2003). HES and HERP families: multiple effectors of the Notch signaling pathway. J. Cell. Physiol..

[17] Kobayashi T., Kageyama R. (2014). Expression dynamics and functions of Hes factors in development and diseases. Curr. Top. Dev. Biol..

[18] Iso T., Sartorelli V., Poizat C., Iezzi S., Wu H.Y., Chung G. (2001). HERP, a novel heterodimer partner of HES/E(spl) in Notch signaling. Mol. Cell. Biol..

[19] Hosaka Y., Saito T., Sugita S., Hikata T., Kobayashi H., Fukai A. (2013). Notch signaling in chondrocytes modulates endochondral ossification and osteoarthritis development. Proc. Natl. Acad. Sci. U. S. A..

[20] Zanotti S., Canalis E. (2016). Notch signaling and the skeleton. Endocr. Rev..

[21] Shang Y., Smith S., Hu X. (2016). Role of Notch signaling in regulating innate immunity and inflammation in health and disease. Protein Cell.

[22] Kapoor M., Martel-Pelletier J., Lajeunesse D., Pelletier J.P., Fahmi H. (2011). Role of proinflammatory cytokines in the pathophysiology of osteoarthritis. Nat. Rev. Rheumatol..

[23] Mirando A.J., Liu Z., Moore T., Lang A., Kohn A., Osinski A.M. (2013). RBP-Jkappa-dependent Notch signaling is required for murine articular cartilage and joint maintenance. Arthritis Rheum..

[24] Mead T.J., Yutzey K.E. (2009). Notch pathway regulation of chondrocyte differentiation and proliferation during appendicular and axial skeleton development. Proc. Natl. Acad. Sci. U. S. A..

[25] Zanotti S., Canalis E. (2013). Notch suppresses nuclear factor of activated T cells (NFAT) transactivation and Nfatc1 expression in chondrocytes. Endocrinology.

[26] Sugita S., Hosaka Y., Okada K., Mori D., Yano F., Kobayashi H. (2015). Transcription factor Hes1 modulates osteoarthritis development in cooperation with calcium/calmodulin-dependent protein kinase 2. Proc. Natl. Acad. Sci. U. S. A..

[27] Canalis E., Schilling L., Yee S.P., Lee S.K., Zanotti S. (2016). Hajdu cheney mouse mutants exhibit osteopenia, increased osteoclastogenesis and bone resorption. J. Biol. Chem..

[28] Canalis E. (2018). Clinical and experimental aspects of notch receptor signaling: Hajdu-Cheney syndrome and related disorders. Metabolism.

[29] Isidor B., Lindenbaum P., Pichon O., Bezieau S., Dina C., Jacquemont S. (2011). Truncating mutations in the last exon of NOTCH2 cause a rare skeletal disorder with osteoporosis. Nat. Genet..

[30] Majewski J., Schwartzentruber J.A., Caqueret A., Patry L., Marcadier J., Fryns J.P. (2011). Mutations in NOTCH2 in families with Hajdu-Cheney syndrome. Hum. Mutat..

[31] Simpson M.A., Irving M.D., Asilmaz E., Gray M.J., Dafou D., Elmslie F.V. (2011). Mutations in NOTCH2 cause Hajdu-Cheney syndrome, a disorder of severe and progressive bone loss. Nat. Genet..

[32] Zhao W., Petit E., Gafni R.I., Collins M.T., Robey P.G., Seton M. (2013). Mutations in NOTCH2 in patients with Hajdu-Cheney syndrome. Osteoporos. Int..

[33] Yu J., Canalis E. (2019). The Hajdu Cheney mutation sensitizes mice to the osteolytic actions of tumor necrosis factor alpha. J. Biol. Chem..

[34] Zanotti S., Yu J., Bridgewater D., Wolf J.M., Canalis E. (2018). Mice harboring a Hajdu Cheney Syndrome mutation are sensitized to osteoarthritis. Bone.

[35] von Vopelius E., Oheim R., Amling M., Rolvien T., Beil F.T. (2021). Skeletal characterization in a patient with Hajdu-Cheney syndrome undergoing total knee arthroplasty. Osteoporos. Int..

[36] Zanotti S., Canalis E. (2013). Interleukin 6 mediates select effects of notch in chondrocytes. Osteoarthritis Cartilage.

[37] Gu Q., Yang H., Shi Q. (2017). Macrophages and bone inflammation. J. Orthop. Translat..

[38] Kwan Tat S., Padrines M., Theoleyre S., Heymann D., Fortun Y. (2004). IL-6, RANKL, TNF-alpha/IL-1: interrelations in bone resorption pathophysiology. Cytokine Growth Factor Rev..

[39] Zanotti S., Yu J., Sanjay A., Schilling L., Schoenherr C., Economides A.N. (2017). Sustained Notch2 signaling in osteoblasts, but not in osteoclasts, is linked to osteopenia in a mouse model of Hajdu-Cheney syndrome. J. Biol. Chem..

[40] Capellini T.D., Chen H., Cao J., Doxey A.C., Kiapour A.M., Schoor M. (2017). Ancient selection for derived alleles at a GDF5 enhancer influencing human growth and osteoarthritis risk. Nat. Genet..

[41] Loughlin J. (2015). Genetic contribution to osteoarthritis development: current state of evidence. Curr. Opin. Rheumatol..

[42] Tang J., Su N., Zhou S., Xie Y., Huang J., Wen X. (2016). Fibroblast growth factor receptor 3 inhibits osteoarthritis progression in the knee joints of Adult mice. Arthritis Rheumatol..

[43] Tang Z., Davidson D., Li R., Zhong M.C., Qian J., Chen J. (2021). Inflammatory macrophages exploit unconventional pro-phagocytic integrins for phagocytosis and anti-tumor immunity. Cell Rep..

[44] Shah V.B., Ozment-Skelton T.R., Williams D.L., Keshvara L. (2009). Vav1 and PI3K are required for phagocytosis of beta-glucan and subsequent superoxide generation by microglia. Mol. Immunol..

[45] Xing Q., Feng Y., Sun H., Yang S., Sun T., Guo X. (2021). Scavenger receptor MARCO contributes to macrophage phagocytosis and clearance of tumor cells. Exp. Cell Res..

[46] Roskar S., Hafner-Bratkovic I. (2022). The role of Inflammasomes in osteoarthritis and secondary joint Degeneration diseases. Life (Basel).

[47] Ridley A.J. (2001). Rho family proteins: coordinating cell responses. Trends Cell Biol..

[48] Bruunsgaard H., Skinhoj P., Pedersen A.N., Schroll M., Pedersen B.K. (2000). Ageing, tumour necrosis factor-alpha (TNF-alpha) and atherosclerosis. Clin. Exp. Immunol..

[49] Provinciali M., Barucca A., Cardelli M., Marchegiani F., Pierpaoli E. (2010). Inflammation, aging, and cancer vaccines. Biogerontology.

[50] Franceschi C., Capri M., Monti D., Giunta S., Olivieri F., Sevini F. (2007). Inflammaging and anti-inflammaging: a systemic perspective on aging and longevity emerged from studies in humans. Mech. Ageing Dev..

[51] Wu D., Ren Z., Pae M., Guo W., Cui X., Merrill A.H. (2007). Aging up-regulates expression of inflammatory mediators in mouse adipose tissue. J. Immunol..

[52] Liu Z., Ren Y., Mirando A.J., Wang C., Zuscik M.J., O'Keefe R.J. (2016). Notch signaling in postnatal joint chondrocytes, but not subchondral osteoblasts, is required for articular cartilage and joint maintenance. Osteoarthritis Cartilage.

[53] Liao Y., Ren Y., Luo X., Mirando A.J., Long J.T., Leinroth A. (2022). Interleukin-6 signaling mediates cartilage degradation and pain in posttraumatic osteoarthritis in a sex-specific manner. Sci. Signal..

[54] Fukushima H., Nakao A., Okamoto F., Shin M., Kajiya H., Sakano S. (2008). The association of Notch2 and NF-kappaB accelerates RANKL-induced osteoclastogenesis. Mol. Cell. Biol..

[55] Natoli G., Chiocca S. (2008). Nuclear ubiquitin ligases, NF-kappaB degradation, and the control of inflammation. Sci. Signal..

[56] Nie L., Wu H., Sun X.H. (2008). Ubiquitination and degradation of Tal1/SCL are induced by notch signaling and depend on Skp2 and CHIP. J. Biol. Chem..

[57] Nie L., Xu M., Vladimirova A., Sun X.H. (2003). Notch-induced E2A ubiquitination and degradation are controlled by MAP kinase activities. EMBO J..

[58] Tang J., Zhan M.N., Yin Q.Q., Zhou C.X., Wang C.L., Wo L.L. (2017). Impaired p65 degradation by decreased chaperone-mediated autophagy activity facilitates epithelial-to-mesenchymal transition. Oncogenesis.

[59] Jiao K., Zhang J., Zhang M., Wei Y., Wu Y., Qiu Z.Y. (2013). The identification of CD163 expressing phagocytic chondrocytes in joint cartilage and its novel scavenger role in cartilage degradation. PLoS One.

[60] Zhou C., Zheng H., Buckwalter J.A., Martin J.A. (2016). Enhanced phagocytic capacity endows chondrogenic progenitor cells with a novel scavenger function within injured cartilage. Osteoarthritis Cartilage.

[61] Fujimura S., Jiang Q., Kobayashi C., Nishinakamura R. (2010). Notch2 activation in the embryonic kidney depletes nephron progenitors. J. Am. Soc. Nephrol..

[62] Lee S.Y., Long F. (2018). Notch signaling suppresses glucose metabolism in mesenchymal progenitors to restrict osteoblast differentiation. J. Clin. Invest..

[63] Han H., Tanigaki K., Yamamoto N., Kuroda K., Yoshimoto M., Nakahata T. (2002). Inducible gene knockout of transcription factor recombination signal binding protein-J reveals its essential role in T versus B lineage decision. Int. Immunol..

[64] Pfander D., Cramer T., Schipani E., Johnson R.S. (2003). HIF-1alpha controls extracellular matrix synthesis by epiphyseal chondrocytes. J. Cell Sci..

[65] Iso T., Sartorelli V., Chung G., Shichinohe T., Kedes L., Hamamori Y. (2001). HERP, a new primary target of Notch regulated by ligand binding. Mol. Cell. Biol..

[66] Nakagawa O., Nakagawa M., Richardson J.A., Olson E.N., Srivastava D. (1999). HRT1, HRT2, and HRT3: a new subclass of bHLH transcription factors marking specific cardiac, somitic, and pharyngeal arch segments. Dev. Biol..

[67] Kutyavin I.V., Afonina I.A., Mills A., Gorn V.V., Lukhtanov E.A., Belousov E.S. (2000). 3'-minor groove binder-DNA probes increase sequence specificity at PCR extension temperatures. Nucleic Acids Res..

[68] Gibson D.G., Young L., Chuang R.Y., Venter J.C., Hutchison C.A., Smith H.O. (2009). Enzymatic assembly of DNA molecules up to several hundred kilobases. Nat. Methods.

[69] Kouadjo K.E., Nishida Y., Cadrin-Girard J.F., Yoshioka M., St-Amand J. (2007). Housekeeping and tissue-specific genes in mouse tissues. BMC Genomics.

[70] Kim D., Langmead B., Salzberg S.L. (2015). HISAT: a fast spliced aligner with low memory requirements. Nat. Methods.

[71] Anders S., Pyl P.T., Huber W. (2015). HTSeq--a Python framework to work with high-throughput sequencing data. Bioinformatics.

[72] Love M.I., Huber W., Anders S. (2014). Moderated estimation of fold change and dispersion for RNA-seq data with DESeq2. Genome Biol..

[73] Henkel T., Ling P.D., Hayward S.D., Peterson M.G. (1994). Mediation of Epstein-Barr virus EBNA2 transactivation by recombination signal-binding protein J kappa. Science.

[74] Zanotti S., Canalis E. (2017). Parathyroid hormone inhibits Notch signaling in osteoblasts and osteocytes. Bone.

[75] Zanotti S., Smerdel-Ramoya A., Canalis E. (2013). Nuclear factor of activated T-cells (Nfat)c2 inhibits notch signaling in osteoblasts. J. Biol. Chem..

[76] Sisto M., Lisi S., D'Amore M., Lofrumento D.D. (2014). Rituximab-mediated Raf kinase inhibitor protein induction modulates NF-kappaB in Sjogren syndrome. Immunology.

