# Supplementary material for: NOTCH2 sensitizes the chondrocyte to the inflammatory response of tumor necrosis factor α
Source: J Biol Chem. 2023 Oct 20;299(12):105372. doi: 10.1016/j.jbc.2023.105372 (PMC10692730; doi:10.1016/j.jbc.2023.105372)

NOTCH2 Sensitizes the Chondrocyte to the Inflammatory Response of Tumor Necrosis Factor α

Canalis, E.*[1,2,3], Yu, J.[1,3], Singh, V.[4], Mocarska, M.[3] and Schilling, L.[3]

Departments of [1]Orthopaedic Surgery, [2]Medicine, the [3]UConn Musculoskeletal Institute
UConn Health, Farmington, CT 06030
and [4]Computational Biology Core, Institute for System Genomics, UConn, Storrs, CT 06269

Supporting Information:

- **Table S1.** Two hundred and eight genes were differentially regulated (log2fc1 or greater p<0.05) between *Notch2$^{tm1.1Ecan}$* and control chondrocytes.
- **Figure S1.** TNFα decreases RBPJκ binding to DNA consensus sequences in chondrocytes.
- **Figure S2**. The phagosome formation and osteoarthritis pathways are enhanced in *Notch2$^{tm1.1Ecan}$* chondrocytes.
- **Figure S3**. The osteoarthritis and phagosome formation pathways are influenced in *Notch2$^{tm1.1Ecan}$* mutant chondrocytes.

Table S1. Two hundred and eight genes were differentially regulated (log2fc1 or greater *p*<0.05) between *Notch2*$^{tm1.1Ecan}$ and control chondrocytes.

Gene	ENSEMBL	Base Mean	Fold Change	Normalized Counts							
				Notch2	Notch2	Notch2	Notch2	Control	Control	Control	Control
Marco	ENSMUSG00000026390	29.42910614	2.185959766	64.91537766	48.86372622	57.88783474	19.79177902	14.17743767	8.581401796	14.77150588	6.443786107
Gzmc	ENSMUSG00000079186	32.47194367	2.130095783	57.70255792	52.01622468	45.99307418	55.41698127	8.95417116	10.9217841	12.66129075	16.10946527
Ccl19	ENSMUSG00000071005	26.79828066	2.09382237	40.57211104	33.88935851	38.0632338	63.33369288	5.96944744	14.82242128	9.144265543	8.591714809
Adamts15	ENSMUSG00000033453	497.1441777	2.079497426	737.5108184	767.6333764	828.6683193	890.6300561	198.4841274	193.4716041	185.698931	175.0561892
Gzme	ENSMUSG00000022156	146.98746	1.998114374	227.2038218	242.7423819	213.3127061	257.2931273	38.80140836	57.72943027	64.71326384	74.10354023
Col6a4	ENSMUSG00000032572	38.55816134	1.910705812	71.22659493	59.89747085	51.54396244	59.37533707	15.66979953	17.94293103	7.034050417	25.77514443
Gzmd	ENSMUSG00000059256	22.80626816	1.874585962	41.47371351	35.46560774	34.89129765	31.66684644	14.9236186	10.9217841	7.737455459	5.369821756
Neb	ENSMUSG00000026950	28.47042766	1.855945208	55.89935299	24.43186311	41.23516995	59.37533707	13.43125674	9.361529232	17.58512604	6.443786107
C1qb	ENSMUSG00000036905	85.53314443	1.830198408	138.84678	130.0405617	117.3616376	150.4175206	29.8472372	35.88586206	46.42473276	35.44082359
Actc1	ENSMUSG00000068614	15.11777613	1.81736391	27.94967649	19.70311541	16.65266479	31.66684644	9.70035209	4.680764616	8.440860501	2.147928702
Arhgap30	ENSMUSG00000048865	26.19478399	1.791246778	31.55608636	51.22810007	36.47726573	43.54191385	8.95417116	10.14165667	14.77150588	12.88757221
Stmn2	ENSMUSG00000027500	26.23818397	1.659609734	34.26089377	29.16061081	36.47726573	63.33369288	16.41598046	13.26216641	10.55107563	6.443786107
Vav1	ENSMUSG00000034116	14.65969019	1.649102326	20.73685675	34.67748312	21.41056901	11.87506741	11.93889488	4.680764616	11.95788571	0
Adamts16	ENSMUSG00000049538	33.56225853	1.611866348	45.98172584	59.89747085	43.61412207	51.45862546	11.19271395	21.06344077	8.440860501	26.84910878
Rac2	ENSMUSG00000033220	36.27217599	1.595214344	55.89935299	66.20246778	42.82113803	51.45862546	12.68507581	15.60254872	17.58512604	27.92307313
Olfm2	ENSMUSG00000032172	45.6198094	1.584438012	64.91537766	53.59247392	80.09138779	75.20876029	14.9236186	18.72305846	27.43279663	30.07100183
Serpina3h	ENSMUSG00000041449	59.50164156	1.56419727	90.16024675	85.11745857	91.19316432	91.04218351	45.51703673	35.10573462	23.91577142	13.96153657
Pi16	ENSMUSG00000024011	20.11859414	1.563624905	40.57211104	25.21998773	19.82460094	35.62520224	14.9236186	14.82242128	3.517025209	6.443786107
Mcpt8	ENSMUSG00000022157	45.09886109	1.561711827	48.68653325	56.74497238	55.50888263	114.7923183	20.14688511	23.40382308	21.10215125	20.40532267
Fcer1g	ENSMUSG00000058715	52.86145443	1.549161779	64.91537766	83.54120934	78.50541972	91.04218351	27.60869441	24.18395052	33.763442	19.33135832
Ncf1	ENSMUSG00000015950	40.99634104	1.541364222	67.62018506	56.74497238	67.40364319	51.45862546	20.89306604	16.38267616	28.13620167	19.33135832
Ptprn	ENSMUSG00000026204	306.936002	1.509818254	418.3435449	453.9597791	455.9658216	490.8361198	170.875433	145.1037031	139.9776033	180.426011
Lilrb4b	ENSMUSG00000112023	54.28941407	1.507953326	90.16024675	80.38871087	82.47033991	67.29204868	44.7708558	20.28331334	25.3225815	23.62721573
Myh3	ENSMUSG00000020908	79.11584763	1.506219774	117.2083208	64.62621855	102.2949408	186.0427228	60.44065533	14.04229385	48.53494788	39.73668099
Hcls1	ENSMUSG00000022831	56.46347032	1.499954948	79.34101714	74.87183856	78.50541972	102.9172509	25.37015162	28.86471513	25.3225815	36.51478794
Gm21104	ENSMUSG00000118633	129.8792969	1.498132277	196.5493379	169.4467925	202.2109296	201.876146	69.39482649	73.33197899	68.23028905	57.99407496
Adgre1	ENSMUSG00000004730	154.5027279	1.46443925	259.6615106	232.4967618	202.2109296	209.7928576	92.52643532	74.89223386	73.15412434	91.28696985
Eln	ENSMUSG00000029675	2148.940707	1.445908197	3448.629438	3378.690231	3305.157469	2422.513752	1181.204412	1238.062241	1107.862941	1109.405175

Gene	ENSEMBL	Base Mean	Fold Change	Normalized Counts							
				Notch2	Notch2	Notch2	Notch2	Control	Control	Control	Control
Col6a6	ENSMUSG00000043719	198.5374362	1.408954795	318.265671	216.7342695	310.0567587	308.7517528	92.52643532	94.39541976	109.0277815	138.5414013
C1qc	ENSMUSG00000036896	95.29007824	1.404588977	142.4531899	133.1930602	122.1195418	158.3342322	45.51703673	46.80764616	56.97580838	56.92011061
C1qa	ENSMUSG00000036887	73.62127156	1.402933736	145.1579973	129.2524371	91.98614836	55.41698127	35.07050371	37.44611693	52.75537813	41.8846097
Flt1	ENSMUSG00000029648	729.499013	1.400239738	1094.545396	989.0963936	1059.426674	1088.547846	398.4606166	394.7444826	362.2535965	448.9170988
Lgr5	ENSMUSG00000020140	155.8047497	1.399818664	221.794207	231.7086372	202.2109296	249.3764157	72.37955021	48.36790103	135.7571731	84.84318374
Aoc3	ENSMUSG00000019326	342.1525699	1.39585817	448.0964264	483.9085145	529.7133371	526.461322	185.7990516	166.1671439	216.6487529	180.426011
Pik3ap1	ENSMUSG00000025017	53.06163749	1.393248091	82.94742701	75.65996318	91.19316432	55.41698127	34.32432278	26.52433282	35.87365713	22.55325137
Mfap4	ENSMUSG00000042436	135.7688094	1.390022436	206.4669651	185.9974095	177.6284244	217.7095693	65.66392184	79.57299847	66.12007392	86.99111245
Itgal	ENSMUSG00000030830	20.90423287	1.389689328	38.7689061	35.46560774	39.64920188	3.958355805	15.66979953	8.581401796	15.47491092	9.665679161
Slc43a1	ENSMUSG00000027075	18.8628291	1.376356691	22.54006169	32.31310927	39.64920188	11.87506741	8.20799023	9.361529232	14.06810083	12.88757221
Mfap5	ENSMUSG00000030116	1137.49057	1.369531503	1471.415227	1415.471811	1320.318423	2363.138415	624.5534384	632.6833506	597.8942855	674.4496125
Adh1	ENSMUSG00000074207	28.19675376	1.360190226	26.14647156	33.88935851	38.85621784	67.29204868	11.19271395	18.72305846	13.36469579	16.10946527
Nrros	ENSMUSG00000052384	19.65514606	1.354810248	30.6544839	23.64373849	30.92637746	27.70849063	8.20799023	10.9217841	13.36469579	11.81360786
Serpina3g	ENSMUSG00000041481	258.4617411	1.353235881	392.1970734	396.4266821	334.6392639	360.2103782	155.2056334	161.4863793	113.9516168	153.5769022
Clec4n	ENSMUSG00000023349	15.95815563	1.349540485	22.54006169	25.21998773	16.65266479	27.70849063	11.93889488	5.460892052	6.330645376	11.81360786
Gm29650	ENSMUSG00000099876	16.20835738	1.340737868	21.63845922	20.49124003	17.44564883	35.62520224	11.19271395	6.241019488	8.440860501	8.591714809
Fermt3	ENSMUSG00000024965	29.09937307	1.33187978	48.68653325	40.19435544	38.0632338	39.58355805	14.9236186	18.72305846	17.58512604	15.03550092
Dpep1	ENSMUSG00000019278	224.8276797	1.322663906	369.6570117	306.5804758	300.5409502	308.7517528	127.596939	138.0825562	137.8673882	109.5443638
Gm11816	ENSMUSG00000086233	21.27798056	1.314810283	24.34326662	26.79623696	29.34040939	43.54191385	10.44653302	7.80127436	18.28853109	9.665679161
Hk3	ENSMUSG00000025877	48.21616916	1.310865852	78.43941467	85.90558319	62.64573897	43.54191385	35.07050371	21.84356821	23.91577142	34.36685924
Mpeg1	ENSMUSG00000046805	220.7684163	1.308664398	371.4602166	335.7410866	325.9164394	213.7512135	152.2209097	124.8203898	106.9175663	135.3195082
Fbn1	ENSMUSG00000027204	22865.50835	1.302084474	34168.02871	28146.29443	34741.42367	33087.89617	9975.692853	10309.38407	13898.58022	18596.76671
Laptm5	ENSMUSG00000028581	105.9191226	1.300119028	146.0595997	161.5655464	127.67043	170.2092996	62.67919812	43.68713642	71.04390922	64.43786107
Ptprc	ENSMUSG00000026395	21.46058637	1.293280421	26.14647156	29.94873542	30.13339343	35.62520224	10.44653302	7.021146924	11.95788571	20.40532267
Cybb	ENSMUSG00000015340	240.9844668	1.29212934	339.0025278	331.8004635	348.9129765	348.3353108	162.6674427	123.2601349	118.172047	155.7248309
Fcgr2b	ENSMUSG00000026656	17.10915251	1.291768393	21.63845922	21.27936464	17.44564883	39.58355805	11.93889488	9.361529232	7.034050417	8.591714809
Aldh1a2	ENSMUSG00000013584	400.2184839	1.290399372	595.0576286	570.6022223	609.0117408	486.877764	215.6462888	269.1439654	197.6568167	257.7514443
Ly9	ENSMUSG00000004707	22.27745906	1.279788607	26.14647156	31.52498466	36.47726573	31.66684644	17.16216139	8.581401796	10.55107563	16.10946527
Clec12a	ENSMUSG00000053063	20.659637	1.275600104	29.75288143	41.77060467	20.61758498	23.75013483	12.68507581	13.26216641	10.55107563	12.88757221
Ctsc	ENSMUSG00000030560	165.7365992	1.271281406	243.4326662	232.4967618	229.9653709	229.5846367	98.49588276	99.07618437	86.51882013	106.3224708
Serping1	ENSMUSG00000023224	1157.792454	1.26508547	1518.298555	1508.470516	1613.722516	1919.802565	737.2267588	688.0723986	611.9623863	664.7839334

Gene	ENSEMBL	Base Mean	Fold Change	Normalized Counts							
				Notch2	Notch2	Notch2	Notch2	Control	Control	Control	Control
Dock2	ENSMUSG00000020143	18.99912869	1.254005565	22.54006169	36.25373235	17.44564883	31.66684644	9.70035209	7.80127436	14.77150588	11.81360786
Gper1	ENSMUSG00000053647	54.58297108	1.251263308	84.75063195	72.50746471	72.16154742	79.16711609	32.83196092	37.44611693	30.94982184	26.84910878
Ms4a7	ENSMUSG00000024672	27.48474173	1.251216533	36.0640987	33.88935851	42.82113803	43.54191385	15.66979953	16.38267616	19.69534117	11.81360786
Trpm3	ENSMUSG00000052387	38.19893915	1.250249835	44.17852091	52.8043493	49.16501033	71.25040448	18.65452325	22.62369564	18.99193613	27.92307313
Sorcs1	ENSMUSG00000043531	25.87939221	1.244063034	36.96570117	36.25373235	43.61412207	27.70849063	5.96944744	17.94293103	24.61917646	13.96153657
C5ar1	ENSMUSG00000049130	38.42353828	1.243155329	57.70255792	63.83809393	32.51234554	63.33369288	19.40070418	19.5031859	25.3225815	25.77514443
Csf1r	ENSMUSG00000024621	202.0819915	1.240775748	267.7759329	271.9029927	286.2672376	312.7101086	114.1656823	102.1966941	123.0958823	138.5414013
Serpina3e-ps	ENSMUSG00000091553	18.84707962	1.232550829	18.93365182	30.73686004	34.89129765	19.79177902	11.19271395	16.38267616	7.034050417	11.81360786
Dkk2	ENSMUSG00000028031	59.13920428	1.232514585	76.63620974	96.93932782	91.98614836	63.33369288	29.10105627	34.32560718	46.42473276	34.36685924
Slc13a3	ENSMUSG00000018459	16.10585214	1.231952677	25.24486909	22.85561388	15.85968075	27.70849063	6.71562837	19.5031859	7.737455459	3.221893054
Slamf7	ENSMUSG00000038179	16.81081571	1.228798852	23.44166416	26.00811234	23.78952113	19.79177902	8.20799023	10.14165667	9.144265543	13.96153657
Cd180	ENSMUSG00000021624	23.30034161	1.227625107	23.44166416	43.3468539	25.3754892	39.58355805	14.9236186	11.70191154	14.06810083	13.96153657
Ncf2	ENSMUSG00000026480	25.63438268	1.22506102	34.26089377	38.6181062	41.23516995	27.70849063	13.43125674	17.16280359	15.47491092	17.18342962
Asic3	ENSMUSG00000038276	197.9620806	1.224704862	296.6272118	240.378008	247.4110197	332.5018876	125.3583962	120.9197526	101.290326	119.210043
Cdh5	ENSMUSG00000031871	15.69002706	1.222417232	32.45768883	21.27936464	11.10177653	23.75013483	12.68507581	9.361529232	8.440860501	6.443786107
Efhd1	ENSMUSG00000026255	18.46119385	1.22233602	26.14647156	29.94873542	26.16847324	19.79177902	9.70035209	16.38267616	7.737455459	11.81360786
Syngr1	ENSMUSG00000022415	14.97054035	1.211859559	27.04807403	20.49124003	16.65266479	19.79177902	12.68507581	8.581401796	9.144265543	5.369821756
Kng2	ENSMUSG00000060459	55.03949226	1.211491648	69.42339	75.65996318	75.33348357	87.0838277	30.59341813	29.64484257	25.3225815	47.25443145
Asxl3	ENSMUSG00000045215	31.06587661	1.193589951	36.0640987	40.98248005	55.50888263	39.58355805	22.3854279	16.38267616	18.28853109	19.33135832
Col4a1	ENSMUSG00000031502	10598.32986	1.189380109	14976.51859	14865.60651	16049.99692	13022.9906	6211.956242	6173.928529	6717.518149	6768.123341
Ms4a6d	ENSMUSG00000024679	18.33528712	1.187187633	28.85127896	22.85561388	29.34040939	19.79177902	10.44653302	10.9217841	12.66129075	11.81360786
Nlrp3	ENSMUSG00000032691	39.74542001	1.185361558	62.21057026	54.38059853	49.16501033	55.41698127	25.37015162	21.06344077	26.72939159	23.62721573
Rpl34-ps1	ENSMUSG00000068396	21.0111192	1.180579141	30.6544839	29.16061081	19.0316169	39.58355805	13.43125674	4.680764616	17.58512604	13.96153657
Hsd11b1	ENSMUSG00000016194	25.49430843	1.174922466	29.75288143	45.71122775	26.16847324	39.58355805	8.95417116	17.16280359	14.06810083	22.55325137
Vgf	ENSMUSG00000037428	31.37135857	1.17430818	43.27691844	40.98248005	39.64920188	51.45862546	23.87778976	10.9217841	20.39874621	20.40532267
Ifitm1	ENSMUSG00000025491	46.86772712	1.165737697	63.11217273	52.8043493	63.438723	83.1254719	35.07050371	23.40382308	23.91577142	30.07100183
Clec4e	ENSMUSG00000030142	33.19906977	1.16556084	53.19454558	46.49935237	43.61412207	39.58355805	19.40070418	17.94293103	26.02598654	19.33135832
Apbb1ip	ENSMUSG00000026786	191.6199043	1.163505823	256.9567032	267.9623696	264.8566685	269.1681947	117.150406	99.85631181	121.6890722	135.3195082
Msr1	ENSMUSG00000025044	55.95686574	1.157755788	87.45543935	96.1512032	70.57557934	51.45862546	38.80140836	39.78649924	36.57706217	26.84910878
Myh11	ENSMUSG00000018830	32.36056463	1.153205796	46.88332831	31.52498466	59.47380282	39.58355805	12.68507581	14.82242128	28.13620167	25.77514443
Ptafr	ENSMUSG00000056529	20.57600872	1.152026219	19.83525429	28.37248619	27.75444131	39.58355805	11.93889488	17.94293103	8.440860501	10.73964351

Gene	ENSEMBL	Base Mean	Fold Change	Normalized Counts							
				Notch2	Notch2	Notch2	Notch2	Control	Control	Control	Control
Adamts8	ENSMUSG00000031994	55.82591512	1.145753245	62.21057026	80.38871087	72.16154742	95.00053931	45.51703673	39.0063718	16.881721	35.44082359
Nhsl2	ENSMUSG00000079481	196.7113032	1.14469508	238.9246539	277.419865	363.1866892	193.9594344	120.1351297	128.7210269	118.172047	133.1715795
Serpina3f	ENSMUSG00000066363	66.3318943	1.143891826	89.25864429	111.9136955	90.40018028	71.25040448	53.72502696	53.04866565	28.83960671	32.21893054
Agtr1a	ENSMUSG00000049115	427.4682314	1.138668583	596.8608335	557.9922284	580.4643155	621.4618613	305.1880004	263.6830734	236.344094	257.7514443
Kng1	ENSMUSG00000022875	56.84227506	1.135374881	82.94742701	86.69370781	73.74751549	67.29204868	34.32432278	40.56662667	35.87365713	33.29289489
Negr1	ENSMUSG00000040037	73.82681374	1.128214161	97.37306649	101.6680755	107.0528451	98.95889512	46.26321766	46.02751873	49.23835292	44.0325384
Lama2	ENSMUSG00000019899	215.8195272	1.128093077	311.9544538	256.1405003	347.3270085	265.2098389	92.52643532	101.4165667	175.8512604	176.1301536
Apoe	ENSMUSG00000002985	1749.80628	1.12784585	2320.724751	2308.417001	2279.829108	2719.390438	1040.176216	1095.29892	1178.90685	1055.706957
Thsd7a	ENSMUSG00000032625	136.9777884	1.124333672	201.0573503	189.1499079	194.2810892	162.292588	72.37955021	66.31083206	108.3243764	102.0266134
Kcnf1	ENSMUSG00000051726	279.9364174	1.122849962	363.3457944	425.5872929	417.1096038	316.6684644	166.3983474	170.8479085	176.5546655	202.9792624
Il6	ENSMUSG00000025746	16.48881561	1.122083888	14.42563948	22.06748926	34.09831362	19.79177902	13.43125674	9.361529232	13.36469579	5.369821756
Tlr13	ENSMUSG00000033777	20.57593653	1.120950551	30.6544839	24.43186311	23.78952113	35.62520224	11.19271395	10.9217841	16.17831596	11.81360786
Fat3	ENSMUSG00000074505	44.0494127	1.119959245	64.91537766	34.67748312	79.29840376	63.33369288	19.40070418	28.0845877	37.98387225	24.70118008
Mycl	ENSMUSG00000028654	28.23831581	1.118773427	36.0640987	45.71122775	45.99307418	23.75013483	23.87778976	12.48203898	15.47491092	22.55325137
Tyrobp	ENSMUSG00000030579	47.10381781	1.114001867	66.7185826	66.20246778	45.20009014	83.1254719	22.3854279	34.32560718	30.94982184	27.92307313
Ubd	ENSMUSG00000035186	31.38671477	1.106258038	34.26089377	52.01622468	38.85621784	47.50026966	17.90834232	26.52433282	18.99193613	15.03550092
Nav3	ENSMUSG00000020181	31.21629702	1.099913605	44.17852091	35.46560774	44.4071061	47.50026966	20.14688511	25.74420539	16.17831596	16.10946527
Gm9913	ENSMUSG00000053615	34.59145062	1.089949832	54.99775052	52.8043493	48.37202629	27.70849063	12.68507581	21.84356821	21.80555629	36.51478794
Stab1	ENSMUSG00000042286	228.9193135	1.087985385	301.1352242	312.0973481	317.193615	316.6684644	173.8601567	143.5434482	131.5367428	135.3195082
Lcp1	ENSMUSG00000021998	222.568835	1.087336078	313.7576587	285.3011111	299.7479662	312.7101086	162.6674427	131.8415367	123.0958823	151.4289735
Ston2	ENSMUSG00000020961	46.72217744	1.083653923	60.40736532	49.65185083	80.09138779	63.33369288	23.13160883	34.32560718	29.54301175	33.29289489
Pirb	ENSMUSG00000058818	35.66664863	1.081909508	61.30896779	40.98248005	39.64920188	51.45862546	17.90834232	13.26216641	25.3225815	35.44082359
Tnfaip8l3	ENSMUSG00000074345	46.54770918	1.080149163	59.50576286	66.99059239	65.02469108	59.37533707	23.87778976	34.32560718	24.61917646	38.66271664
Adamts12	ENSMUSG00000047497	559.7619506	1.07427767	707.757937	734.5321425	768.4015324	831.254719	335.7814185	301.9093177	392.5000133	405.9585247
Myh8	ENSMUSG00000055775	33.55666979	1.07198859	52.29294312	32.31310927	47.57904225	51.45862546	27.60869441	10.14165667	30.94982184	16.10946527
Cldn15	ENSMUSG00000001739	19.62937371	1.0705207	27.94967649	25.21998773	28.54742535	23.75013483	11.93889488	14.04229385	10.55107563	15.03550092
Fabp7	ENSMUSG00000019874	36.54903752	1.066999153	59.50576286	59.10934623	30.92637746	47.50026966	20.14688511	22.62369564	22.50896134	30.07100183
Ccl3	ENSMUSG00000000982	68.63087173	1.063362305	109.995501	77.23621241	73.74751549	114.7923183	42.53231301	45.24739129	43.61111259	41.8846097
Trem2	ENSMUSG00000023992	45.49546863	1.06175109	55.89935299	46.49935237	47.57904225	102.9172509	26.86251348	24.18395052	26.72939159	33.29289489
Col15a1	ENSMUSG00000028339	603.0779201	1.055876134	863.7351639	801.5227349	822.324447	763.9626703	370.8519222	404.8861393	415.0089746	382.331309
En1	ENSMUSG00000058665	31.46914049	1.054761213	40.57211104	48.86372622	36.47726573	43.54191385	18.65452325	20.28331334	17.58512604	25.77514443

Gene	ENSEMBL	Base Mean	Fold Change	Normalized Counts							
				Notch2	Notch2	Notch2	Notch2	Control	Control	Control	Control
Myo1f	ENSMUSG00000024300	52.68971238	1.052267345	77.53781221	79.60058626	68.98961127	55.41698127	31.33959906	32.76535231	41.50089746	34.36685924
Kif26b	ENSMUSG00000026494	575.6232504	1.049380842	690.6274901	750.2946348	788.2261333	878.7549886	328.3196092	363.5393852	314.4220537	490.8017085
Rpl28-ps1	ENSMUSG00000058603	17.92632461	1.048664455	26.14647156	19.70311541	22.99653709	27.70849063	11.19271395	12.48203898	4.923835292	18.25739397
Casp1	ENSMUSG00000025888	20.30850248	1.046148628	34.26089377	22.85561388	22.20355305	31.66684644	16.41598046	11.70191154	14.77150588	8.591714809
Cfd	ENSMUSG00000061780	209.5274434	1.043764544	216.3845922	301.0636035	280.7163493	336.4602434	102.9729683	145.8838305	145.6048436	147.1331161
Lcp2	ENSMUSG00000002699	26.24114791	1.042106008	36.96570117	26.79623696	29.34040939	51.45862546	18.65452325	17.94293103	12.66129075	16.10946527
Mmp17	ENSMUSG00000029436	115.2518443	1.037091984	163.1900466	177.3280387	137.9792225	138.5424532	80.58754044	68.65121437	79.48476972	76.25146893
Crabp1	ENSMUSG00000032291	68.74765444	1.033833253	83.84902948	71.71934009	96.74405258	122.7090299	32.83196092	62.41019488	46.42473276	33.29289489
Acod1	ENSMUSG00000022126	242.5514315	1.03282121	311.9544538	291.6061081	306.0918385	407.7106479	182.0681469	144.3235757	166.7069949	129.9496865
Igsf6	ENSMUSG00000035004	32.58709554	1.031607391	45.08012338	55.95684777	39.64920188	31.66684644	20.14688511	20.28331334	23.21236638	24.70118008
Tnfaip6	ENSMUSG00000053475	316.1160305	1.030003321	424.6547622	407.4604267	406.0078272	463.1276292	222.3619171	209.8542803	166.7069949	228.7544068
Fblim1	ENSMUSG00000006219	139.4759298	1.026357806	174.0092762	168.6586679	181.5933446	233.5429925	83.57226416	87.37427283	109.7311865	77.32543329
Postn	ENSMUSG00000027750	6738.9306	1.025937955	8936.683658	9080.77183	9244.60791	8882.550426	4543.495683	4280.559241	4293.584375	4649.191676
C3ar1	ENSMUSG00000040552	49.00391916	1.024042429	73.93140234	61.47372008	80.09138779	43.54191385	40.29377022	28.86471513	33.763442	30.07100183
Gm4468	ENSMUSG00000092014	18.68027449	1.02264707	26.14647156	21.27936464	22.20355305	31.66684644	17.16216139	8.581401796	8.440860501	13.96153657
Acta2	ENSMUSG00000035783	14989.85729	1.019449014	19113.97231	19077.34446	18508.24744	23631.38415	9327.261625	9146.21406	11260.81131	9853.622922
Scg2	ENSMUSG00000050711	137.6219334	1.01835875	196.5493379	167.0824187	176.0424563	201.876146	79.84135951	99.85631181	99.18011089	80.54732634
Csmd1	ENSMUSG00000060924	24.76387155	1.018001978	45.08012338	33.88935851	31.7193615	19.79177902	11.93889488	12.48203898	26.02598654	17.18342962
Cd93	ENSMUSG00000027435	34.39937632	1.017485299	36.0640987	57.533097	42.82113803	47.50026966	23.13160883	15.60254872	24.61917646	27.92307313
Col18a1	ENSMUSG00000001435	4996.032495	1.016462274	6945.94541	6741.617969	7189.193285	5842.533168	3352.590918	3398.235111	3007.759959	3490.384141
Pcdh18	ENSMUSG00000037892	779.7628763	1.014057321	965.6162427	1037.171995	1070.528451	1104.38127	487.2561473	487.5796475	532.4776166	553.0916409
Itgb2	ENSMUSG00000000290	67.82330669	1.013350706	82.94742701	81.17683549	88.81421221	114.7923183	32.83196092	53.04866565	49.23835292	39.73668099
Lgi2	ENSMUSG00000039252	108.8122661	1.013267719	134.3387677	144.2268048	172.0775362	126.6673858	73.12573114	80.35312591	54.86559326	84.84318374
Hic1	ENSMUSG00000043099	310.8516373	1.011427324	376.8698314	398.0029313	468.6535662	419.5857153	208.1844795	185.6703298	225.7930184	204.0532267
Adgrd1	ENSMUSG00000044017	318.7837077	1.010879089	403.016303	450.8072806	411.5587155	439.3774943	184.3066897	205.1735157	222.9793982	233.0502642
Wnt16	ENSMUSG00000029671	65.27042216	1.008305544	69.42339	104.820574	83.26332394	91.04218351	46.26321766	26.52433282	47.1281378	53.69821756
Gvin-ps7	ENSMUSG00000063286	17.98050022	1.005219574	26.14647156	21.27936464	24.58250516	23.75013483	13.43125674	10.14165667	10.55107563	13.96153657
Ebf1	ENSMUSG00000057098	1126.731649	1.002071475	1502.069711	1426.505556	1531.252177	1555.633831	778.26671	677.1506145	738.5752938	804.399299
Hmcn1	ENSMUSG00000066842	308.4865693	1.001440423	451.7028362	377.5116913	511.4747042	300.8350412	145.5052813	155.2453598	215.2419428	310.3756975
Kcp	ENSMUSG00000059022	95.12814547	1.001258236	137.0435751	129.2524371	118.9476056	122.7090299	65.66392184	72.55185155	63.30645376	51.55028886
Atp8b1	ENSMUSG00000039529	82.73891463	1.000413967	97.37306649	124.5236894	98.33002066	122.7090299	52.97884603	46.02751873	57.67921342	62.28993237

Gene	ENSEMBL	Base Mean	Fold Change	Normalized Counts							
				Notch2	Notch2	Notch2	Notch2	Control	Control	Control	Control
Fzd9	ENSMUSG00000049551	405.7279038	-1.0052931	286.7095847	266.3861203	260.8917484	265.2098389	520.0881082	464.1758244	685.1165107	497.2454946
	ENSMUSG00000095041	755.2092106	-1.010292007	533.7486608	479.9678914	545.5730178	439.3774943	1334.171503	1092.17841	782.1864064	834.4703009
Sh3tc2	ENSMUSG00000045629	116.597529	-1.022610822	77.53781221	76.44808779	76.12646761	79.16711609	159.682719	131.0614093	189.9193613	142.8372587
Hapln1	ENSMUSG00000021613	918.4300165	-1.022865327	571.6159644	634.4403162	615.3556131	601.6700823	1127.479385	1068.774587	1569.296648	1158.807535
Cnmd	ENSMUSG00000022025	4949.642295	-1.024861158	3279.128174	3226.58218	3102.946539	3475.436397	6394.024389	6820.654173	6940.497547	6357.868959
Myo5c	ENSMUSG00000033590	37.86831042	-1.030235438	22.54006169	24.43186311	27.75444131	23.75013483	41.78613208	51.48841078	47.83154284	63.36389672
Gchfr	ENSMUSG00000046814	21.36604804	-1.031605344	6.311217273	18.91499079	11.10177653	23.75013483	20.14688511	29.64484257	28.83960671	32.21893054
Mamdc4	ENSMUSG00000026941	23.41644544	-1.032025201	11.72083208	22.85561388	18.23863286	3.958355805	52.2326651	26.52433282	26.02598654	25.77514443
Ppp1r16b	ENSMUSG00000037754	24.38073351	-1.033819156	15.32724195	13.39811848	17.44564883	19.79177902	34.32432278	35.88586206	30.94982184	27.92307313
Acox2	ENSMUSG00000021751	70.81552247	-1.036826515	42.37531597	43.3468539	49.95799437	51.45862546	89.5417116	98.29605694	78.78136468	112.7662569
Tnfrsf25	ENSMUSG00000024793	42.3699394	-1.046539956	31.55608636	26.79623696	20.61758498	35.62520224	78.34899765	62.41019488	49.23835292	34.36685924
Ppp1r1b	ENSMUSG00000061718	568.1336414	-1.046754716	322.7736834	342.8342081	369.5305615	482.9194082	745.4347491	716.1569863	844.7894551	720.6300796
Mir99ahg	ENSMUSG00000090386	29.20760378	-1.05778344	17.13044688	21.27936464	20.61758498	15.83342322	44.02467487	43.68713642	48.53494788	22.55325137
Fgfr3	ENSMUSG00000054252	701.9410711	-1.06283074	424.6547622	435.0447883	432.1763005	530.4196778	762.5969104	786.3684555	1560.152383	684.1152917
Rnf39	ENSMUSG00000036492	50.88189723	-1.068720259	34.26089377	35.46560774	27.75444131	35.62520224	75.36427393	49.14802847	91.44265543	57.99407496
Rwdd2a	ENSMUSG00000032417	18.57000085	-1.080010319	12.62243455	18.91499079	8.722824413	3.958355805	28.35487534	35.88586206	19.69534117	20.40532267
Itgb7	ENSMUSG00000001281	55.17537106	-1.08105865	24.34326662	44.13497852	38.85621784	31.66684644	74.618093	63.97044975	68.23028905	95.58282726
Nog	ENSMUSG00000048616	90.40912029	-1.088283877	61.30896779	63.83809393	65.81767512	31.66684644	112.6733204	105.3172039	189.2159562	93.43489855
Colgalt2	ENSMUSG00000032649	20.1498831	-1.104830461	16.22884442	11.03374463	15.85968075	3.958355805	29.8472372	21.84356821	32.35663192	30.07100183
Tmem88	ENSMUSG00000045377	20.72150196	-1.108375548	11.72083208	10.24562001	15.85968075	15.83342322	21.63924697	27.30446026	30.94982184	32.21893054
Gdf5	ENSMUSG00000038259	316.9751068	-1.127973483	195.6477355	178.1161633	219.6565784	205.8345018	422.3384064	430.6303447	457.2132771	426.3638474
Fbp2	ENSMUSG00000021456	28.11321027	-1.128243384	18.03204935	14.97436771	22.99653709	11.87506741	52.2326651	35.88586206	30.2464168	38.66271664
Matn1	ENSMUSG00000040533	1997.685631	-1.12877962	1401.090235	1221.593155	1158.549679	1231.048655	2731.022204	2642.291626	2934.605834	2661.283662
Syt8	ENSMUSG00000031098	24.62913268	-1.138301408	19.83525429	12.60999386	18.23863286	7.916711609	29.8472372	52.26853821	33.763442	22.55325137
Nptx1	ENSMUSG00000025582	120.468714	-1.138379872	67.62018506	84.32933396	70.57557934	79.16711609	159.682719	174.7485457	136.4605781	191.1656545
Msmp	ENSMUSG00000078719	169.9501763	-1.14568593	133.4371652	100.0918263	84.05630798	106.8756067	237.2855357	248.0805247	212.4283226	237.3461216
Tmem184a	ENSMUSG00000036687	24.19673233	-1.158193239	9.917627143	18.12686618	18.23863286	11.87506741	30.59341813	33.54547975	37.98387225	33.29289489
Kcnk1	ENSMUSG00000033998	24.66127789	-1.160042979	13.52403701	11.82186925	18.23863286	19.79177902	38.05522743	32.76535231	35.17025209	27.92307313
mt-Nd6	ENSMUSG00000064368	97.13359988	-1.165153477	38.7689061	93.78682935	76.91945164	23.75013483	143.2667386	154.4652323	150.5286789	95.58282726
Klk10	ENSMUSG00000030693	43.59731299	-1.172404843	27.04807403	20.49124003	28.54742535	35.62520224	60.44065533	58.5095577	61.19623863	56.92011061
Ano1	ENSMUSG00000031075	71.21232305	-1.172805939	51.39134065	43.3468539	34.09831362	47.50026966	94.01879718	103.756949	73.15412434	122.431936

Gene	ENSEMBL	Base Mean	Fold Change	Normalized Counts							
				Notch2	Notch2	Notch2	Notch2	Control	Control	Control	Control
F13a1	ENSMUSG00000039109	161.478551	-1.210705811	81.14422208	97.72745244	107.0528451	106.8756067	236.5393548	189.570967	219.462373	253.4555869
Entpd3	ENSMUSG00000041608	21.80022581	-1.219612246	21.63845922	11.03374463	11.89476056	3.958355805	28.35487534	31.98522488	38.6872773	26.84910878
Gm44732	ENSMUSG00000108934	27.7484687	-1.226715015	15.32724195	18.91499079	18.23863286	11.87506741	54.47120789	35.10573462	37.98387225	30.07100183
Syn3	ENSMUSG00000059602	22.13118948	-1.231444826	14.42563948	11.82186925	14.27371268	11.87506741	33.57814185	19.5031859	41.50089746	30.07100183
Ucma	ENSMUSG00000026668	426.068489	-1.240395956	293.9224044	250.623628	228.3794028	233.5429925	557.3971547	628.002586	607.7419561	608.9377871
Tnfrsf11b	ENSMUSG00000063727	2341.710907	-1.256859775	1369.534148	1446.208671	1378.999241	1318.132483	3234.694331	3093.205284	3713.97862	3178.93448
Panx3	ENSMUSG00000011118	17.52680095	-1.264997978	6.311217273	7.093121548	16.65266479	11.87506741	20.14688511	14.82242128	42.90770755	20.40532267
Bmp7	ENSMUSG00000008999	19.19684259	-1.267741637	7.21281974	14.97436771	11.10177653	11.87506741	29.10105627	21.84356821	29.54301175	27.92307313
Gdf10	ENSMUSG00000021943	552.7838607	-1.268536142	339.0025278	302.6398527	327.5024075	328.5435318	552.1738882	532.8270388	1455.345031	584.236607
Lctl	ENSMUSG00000032401	67.53273546	-1.272585466	27.94967649	44.13497852	45.99307418	39.58355805	111.1809586	81.91338078	92.84946551	96.65679161
Dancr	ENSMUSG00000106943	15.47517211	-1.287247166	13.52403701	6.304996931	10.30879249	3.958355805	26.86251348	25.74420539	27.43279663	9.665679161
Gdf6	ENSMUSG00000051279	36.0582619	-1.287724476	17.13044688	25.21998773	22.99653709	15.83342322	35.81668464	32.76535231	71.04390922	67.65975412
Tslp	ENSMUSG00000024379	124.5981764	-1.306384	96.47146403	71.71934009	45.20009014	75.20876029	199.9764892	214.5350449	113.2482117	180.426011
Scrg1	ENSMUSG00000031610	117.9903191	-1.30789488	90.16024675	75.65996318	41.23516995	63.33369288	163.4136237	168.5075262	175.1478554	166.4644744
Upb1	ENSMUSG00000033427	23.3681814	-1.313272493	17.13044688	9.457495397	12.6877446	15.83342322	37.3090465	32.76535231	29.54301175	32.21893054
Cntnap3	ENSMUSG00000033063	25.63292506	-1.322821416	18.93365182	13.39811848	13.48072864	11.87506741	32.83196092	40.56662667	47.1281378	26.84910878
Msln	ENSMUSG00000063011	73.41907161	-1.325296215	45.08012338	33.10123389	45.20009014	47.50026966	105.2115111	116.238988	104.8073512	90.2130055
Serpinb6b	ENSMUSG00000042842	19.13260979	-1.345163336	8.114422208	10.24562001	15.06669671	7.916711609	20.14688511	22.62369564	28.13620167	40.81064534
Etohd2	ENSMUSG00000089875	14.98228423	-1.36176198	9.917627143	9.457495397	7.929840376	3.958355805	19.40070418	30.42497	14.06810083	24.70118008
Ncmap	ENSMUSG00000043924	28.80405229	-1.415397809	15.32724195	17.33874156	16.65266479	11.87506741	32.08577999	36.66598949	66.12007392	34.36685924
Usp29	ENSMUSG00000051527	39.19130988	-1.421056649	27.04807403	21.27936464	16.65266479	19.79177902	44.02467487	58.5095577	68.23028905	57.99407496
Xlr3b	ENSMUSG00000073125	14.57534046	-1.42129149	9.917627143	2.364373849	13.48072864	3.958355805	23.87778976	17.16280359	18.99193613	26.84910878
Snorc	ENSMUSG00000026258	35.27428359	-1.426700256	23.44166416	20.49124003	13.48072864	19.79177902	55.21738882	44.46726385	56.97580838	48.3283958
Dpp4	ENSMUSG00000035000	88.80962372	-1.522488726	54.99775052	46.49935237	45.20009014	27.70849063	117.8965869	132.6216641	147.0116537	138.5414013
Angptl7	ENSMUSG00000028989	338.7028771	-1.966378134	142.4531899	130.0405617	127.67043	166.2509438	470.8401668	549.209715	532.4776166	590.6803932

Chondrocytes from newborn *Notch2*$^{tm1.1Ecan}$ mice and control littermates were cultured to confluence and treated with TNFα at 50 ng/ml for 6 hours in the absence of serum. Cells were collected for total RNA extraction and RNASeq data were analyzed using DESeq2 and median of ratios was used to obtain normalized counts. Gene name, Ensembl gene identification, base means, adjusted and individual normalized counts are shown from *Notch2*$^{tm1.1Ecan}$ (Notch2) and control chondrocytes, both treated with TNFα.

Figure S1. **TNFα decreases RBPJκ binding to DNA consensus sequences in chondrocytes.** Chondrocyte-enriched cells from newborn *Notch2*$^{tm1.1Ecan}$ mice and control littermates were cultured to confluence and exposed to TNFα 50 ng/ml for 6 hours or vehicle in the absence of serum. A biotinylated oligonucleotide containing *CSL* or *Rbpj* consensus sequences from the EBNA2 promoter was used. Competition of binding reactions was performed in the presence of unlabeled oligonucleotides containing wild type (WT) homologous or mutant (MT) *CSL* consensus sequences in 200-fold excess. DNA-nuclear protein complexes were resolved by gel electrophoresis, transferred to a nylon membrane, exposed to a streptavidin-horseradish peroxidase conjugate, and visualized by chemiluminescence.

Figure S1

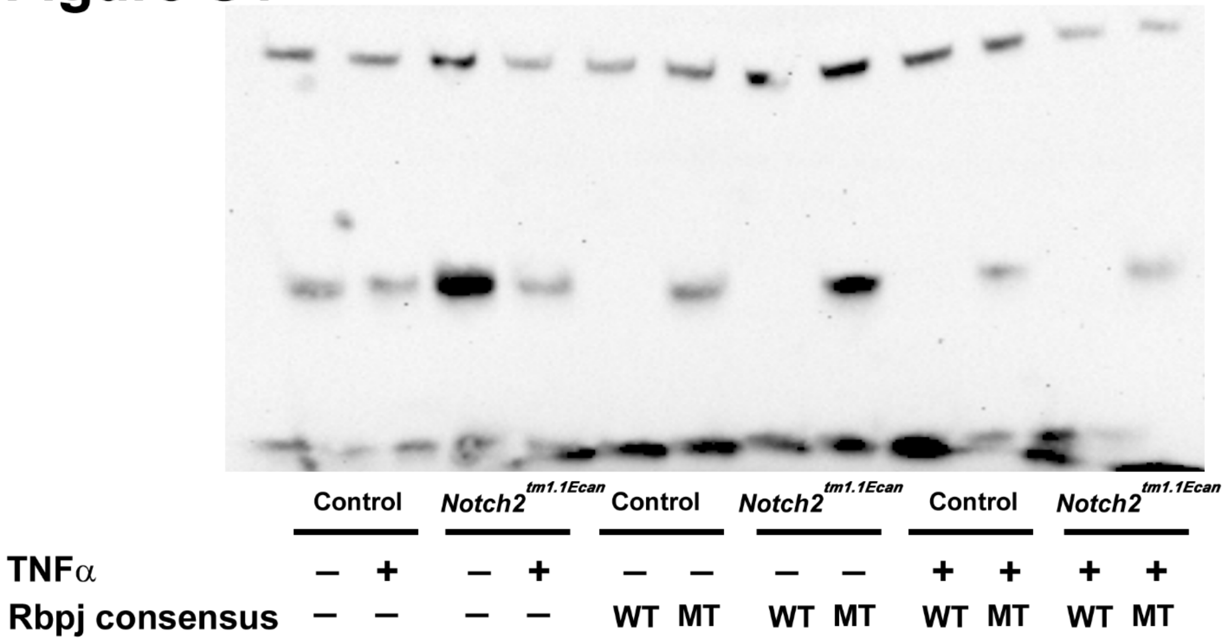

Figure S2. **The phagosome formation and osteoarthritis pathways are enhanced in *Notch2*$^{tm1.1Ecan}$ chondrocytes.** Chondrocyte-enriched cells from newborn *Notch2*$^{em1.1Ecan}$ mice and control littermates were cultured to confluence and treated with TNFα at 50 ng/ml for 6 hours in the absence of serum. Cells were collected for total RNA extraction and analyzed by RNASeq. The Venn diagrams reveal differentially regulated genes and number of genes affected in the phagosome formation and osteoarthritis pathway at log 2fc1 p adjusted value of 0.05.

Figure S2

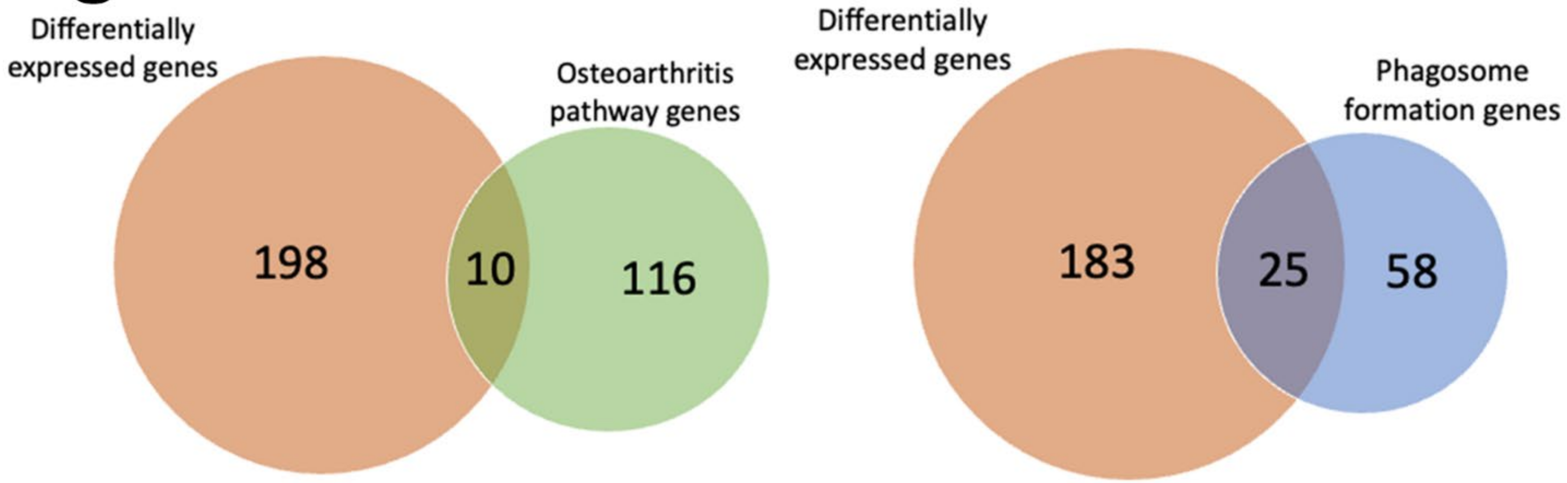

Figure S3. **The osteoarthritis and phagosome formation pathways are influenced in *Notch2$^{tm1.1Ecan}$* mutant chondrocytes.** Chondrocyte-enriched cells from newborn heterozygous *Notch2$^{tm1.1Ecan}$* mice (closed circles, grey bars) and control littermates (open circles, white bars) were cultured to confluence, transferred, and exposed to TNFα at 50 ng/ml or vehicle for 24 hours in the absence of serum and mRNA expression determined by qRT-PCR. Data for mRNA are expressed as relative expression corrected for *Rpl38*. Values are means (bars) ± SD and individual determinations (dots); n = 4 for all data sets. Significantly different between: *control and *Notch2$^{tm1.1Ecan}$*; #TNFα and vehicle, both $p < 0.05$ by two-way ANOVA with post-hoc analysis by Tukey.

Figure S3

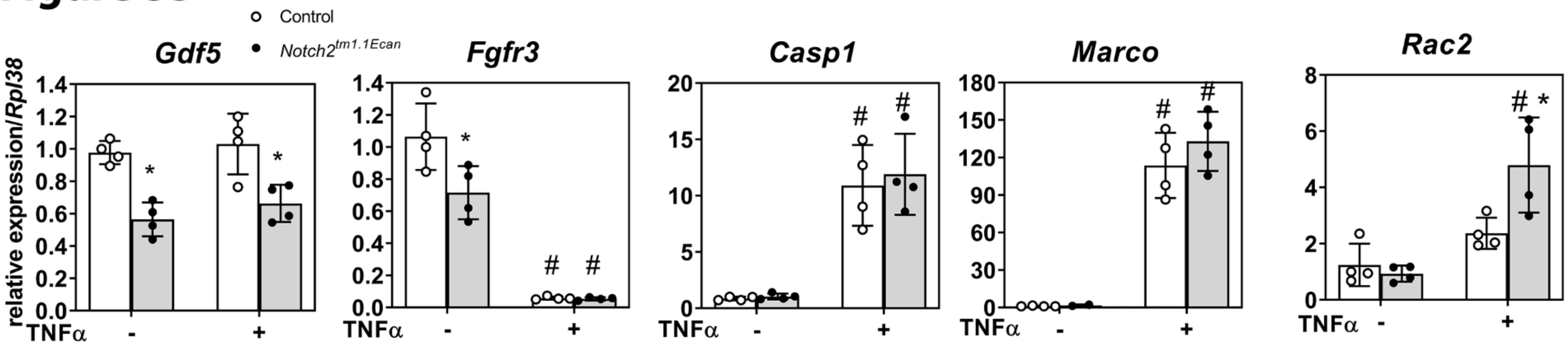

Supplement: Supporting Table S1 and Figures S1–S3 [file mmc1.pdf]
